# Hydrogen Activation by a σσ*-Carbene Through Quantum Tunneling

**DOI:** 10.1021/jacs.5c06016

**Published:** 2025-07-15

**Authors:** Virinder Bhagat, Jan Meisner, J. Philipp Wagner

**Affiliations:** † Institut für Organische Chemie, Eberhard Karls Universität Tübingen, Auf der Morgenstelle 18, 72076 Tübingen, Germany; ‡ Institute for Physical Chemistry, 9170 Heinrich Heine University Düsseldorf, Universitätsstraße 1, 40225 Düsseldorf, Germany; § Institut für Organische und Analytische Chemie, Universität Bremen, Leobener Straße 7, 28359 Bremen, Germany

## Abstract

The electronic structure of carbenes arises from the occupation of a σ and a π frontier orbital. While parent methylene possesses a triplet ground state (σ^1^π^1^), substituents are capable of stabilizing the singlet as the ground state (σ^2^π^0^ or σ^0^π^2^) by altering the frontier orbital energies. Here, we reveal that the 1,2­[I]-shift isomer of 2-iodopyridine, the *N*-iodo Hammick intermediate, features a resonance between its carbene σ and N–I bond σ* orbitals, rendering them frontier orbitals. This singlet carbene is efficiently generated via UV photolysis of 2-iodopyridine in solid neon at 4.4 K and reacts with molecular hydrogen – but not deuterium – via N–I bond cleavage enabled by quantum tunneling. Instanton theory computations demonstrate the preference for a concerted hydrogen addition mechanism at elevated temperatures, while hydrogen atom abstraction dominates below 100 K despite a higher kinetic barrier for this process. Our findings introduce an unprecedented carbene class, unlocking new opportunities for reactivity and electronic structure explorations.

## Graphical abstract

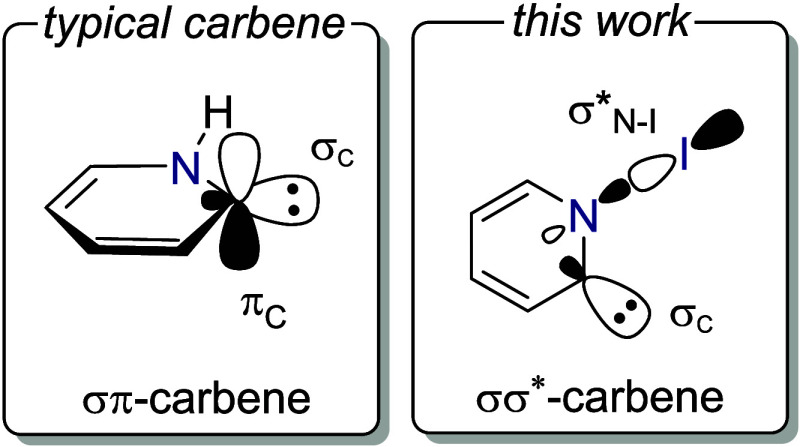

## 1 Introduction

Divalent carbon compounds, namely carbenes, keep fascinating chemists for their electronic structure, in which two nonbonding electrons are distributed over a σ and a π frontier orbital.^1^ Since it became clear from Herzberg’s seminal spectroscopic work that parent methylene, CH_2_, displays a triplet ground state (^3^B_1_ in the bent *C*_2v_ geometry, Figure 1A),^2^ electronic structure theorists have devised strategies to selectively stabilize the singlet through stereoelectronic effects.^3–5^ The low-lying first excited singlet state, ^1^A_1_, with its dominant σ^2^π^0^ electron configuration (Figure 1A) can be favored by lowering the energy of the σ while elevating the π orbital. The former can be stabilized by reducing the carbene angle through incorporation into a cyclic structure or by attaching electronegative atoms to the carbene center. Likewise, the π orbital can be raised in energy by interaction with donor substituents featuring a lone pair or a double bond. All of these structural motifs have been employed in imidazolylidenes (**I**, Figure 1B), a class of N-heterocyclic carbenes (NHCs) first reported by Arduengo,^6^ advancing them to *stable carbenes*.^7^

**Figure 1:**
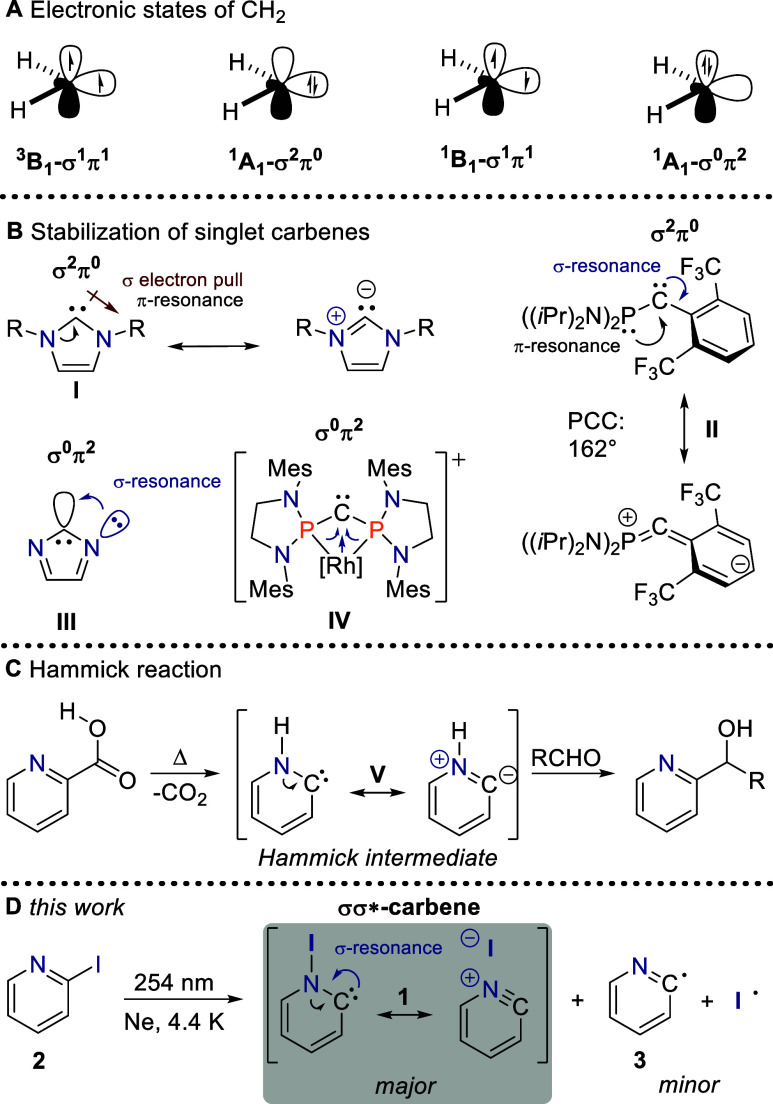
The electronic structure of carbenes. (A) The four lowest electronic states of methylene, CH_2_, featuring a triplet ground state. (B) Resonance interactions stabilizing singlet carbenes. (C) Generation and trapping of the Hammick intermediate, and (D) the *N*-iodo Hammick intermediate reported in this work.

In principle, the energy of the filled σ frontier orbital can also be decreased through resonance interaction with a low-lying vacant orbital of suitable symmetry.^7^ However, such a strategy is less commonly utilized,^8,9^ and has been demonstrated only in a few examples, such as the phosphino­(aryl) carbene **II** (Figure 1B).^10^ In these cases, the stabilization effect becomes evident in the obtuse carbene bond angle. Interestingly, a reverse donor–acceptor interaction involving the empty σ orbital of σ^0^π^2^ carbenes is more commonly employed to achieve the formally doubly excited singlet electron configuration as the ground state.^4^ This type of resonance interaction is observed in matrix isolated carbene **III** ^11^ and metalated carbene **IV**, which is even stable in its crystalline form (Figure 1B).^12^ In this work, we demonstrate that the 1,2­[I]-shift isomer of 2-iodopyridine, the *N*-iodo Hammick intermediate **1**, enjoys a significant resonance stabilization of its carbene σ orbital through interaction with the σ* orbital of the N–I bond (Figure 1D). This stabilization is so profound that these two orbitals become the system’s frontier orbitals.

Pyridin-2-ylidene, **V**, the parent Hammick intermediate, has been proposed as an ylidic transient species in the decarboxylation of α-picolinic acid (Figure 1C).^13,14^ The trapping of **V** with carbonyl compounds results in the formation of alcohols. Subsequent studies recognized that a carbene resonance contributor provides additional stabilization to this intermediate.^3,15^ Experimental detection of **V** in the gas phase *via* neutralization-reionization mass spectrometry revealed its resistance to tautomerization into pyridine on the millisecond time scale.^16^ Further evidence was collected for the presence of a methylated derivative in the reaction of carbon atoms with *N*-methylpyrrol after bimolecular rearrangement.^17^ Although theoretical studies have long suggested that pyridinylidenes can be stabilized through steric protection,^18^ these carbenes were, for many years, only accessible via trapping reactions with reagents such as sulfur^19^ or molecular hydrogen.^20^ It was not until recently that the first stable pyridinylidene, a benzo­[*h*]­isoquinolin-1-ylidene, was successfully crystallized.^21^ Despite the inherent challenge of isolating the 2-carbene isomers of pyridines in pure form, the associated tautomerization can occur spontaneously within the coordination sphere of a metal.^22,23^

We became interested in Hammick intermediates pursuing our work on radical-mediated molecular hydrogen activation. Recently, we demonstrated that the phenyl radical, generated through photolysis of iodobenzene, can cleave molecular hydrogen at cryogenic temperatures *via* quantum mechanical tunneling (QMT).^24^ Building on this, we aimed to photolytically generate the 2-pyridyl radical from 2-iodopyridine (Figure 1D). The latter is expected to display a much reduced reactivity compared to the phenyl radical due to a stabilizing 2 center–3 electron interaction of the neighboring lone-pair with the radical center.^25^ These studies led to the serendipitous discovery of the *N*-iodo Hammick intermediate, **1**, the first of a novel class of σσ*-carbenes.

## 2 Results and Discussion

### 2.1 Trapping the N-iodo Hammick Intermediate

In an attempt to prepare the 2-pyridyl radical (**3**) in matrix isolation, we codeposited the supposed precursor 2-iodopyridine (**2**) with a large excess of neon onto a cold CsI window kept at 4.4 K. Subsequent irradiation of the matrix with λ = 254 nm ultraviolet (UV) light led to the expected bleaching of precursor **2**, which becomes evident from the downward-pointing bands in the difference infrared (IR) spectrum of Figure 2A. While the disappearing bands match well with the computed IR spectrum of 2-iodopyridine (Figure 2D), surprisingly, no such agreement is seen for the upward pointing bands, corresponding to the forming species, with the computed spectrum of the expected photoproduct 2-pyridyl radical (Figure 2C); in particular, a very strong band at 625 cm^–1^ remains unaccounted for. A comparison with literature data reveals that only a small subset of weaker IR bands at 565, 738, and 942 cm^–1^ can be attributed to the target radical **3**.^26^

**Figure 2:**
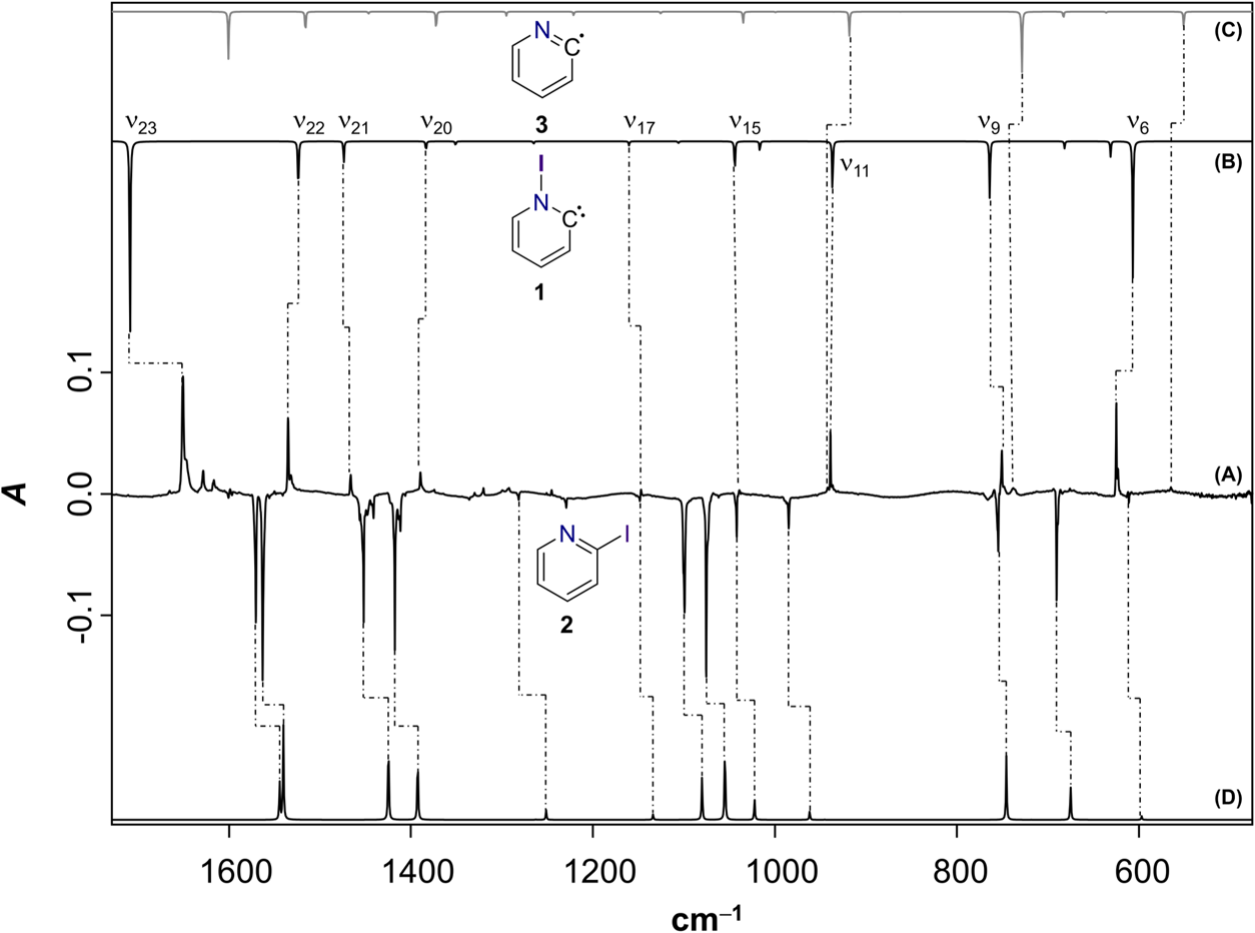
Generation of N-Iodo-Hammick intermediate **1**. (A) Difference IR spectrum after irradiation of a neon matrix containing **2** for 10 min with λ = 254 nm. (B) Anharmonic IR spectrum (fundamental bands only) of **1** computed at the B2PLYP-D3/def2-TZVPP level of theory. (C) and (D) Computed harmonic spectra (scaled, scaling factor (SF): 0.9605) of **3** and **2**, respectively (B2PLYP-D3/def2-TZVPP).

The remaining bands, positioned at 1651, 1535, 1466, 1390, 1147, 1039, 939, 751, and 625 cm^–1^, are much higher in intensity and got bleached at the same rate when the matrix window was exposed to sunlight for 60 min or irradiated with λ = 290–320 nm resulting in the back-formation of precursor **2** (Figure S1). Thus, the set of bands is likely to correspond to a single, photolabile species. We surmised that the iodine migrated to the nitrogen atom upon photolysis of **2**, rendering the observed product an *N*-iodinated version of the Hammick intermediate, **1**. Indeed, the anharmonic IR spectrum of **1** (Figure 2B) computed with the B2PLYP-D3 double-hybrid functional is in superb agreement with the experimental IR spectrum, including the previously unexplained band at 625 cm^–1^.^27^ Thus, we assigned the N-iodo Hammick intermediate **1** as the main spectral carrier.

Our computational explorations with single-reference coupled cluster, DLPNO–CCSD­(T), and multireference perturbation theory, NEVPT2, affirm that there is a significant barrier in excess of 10 kcal mol^–1^ protecting **1** from the back-reaction to iodopyridine, which is more stable than **1** by ∼55–60 kcal mol^–1^ (Figure 3A). While the presence of an N–I bond suggests that **1** can be viewed as a carbene, peculiarly, the two resembling frontier orbitals of this system, dubbed σ and σ* in the following, are both of σ-type featuring contributions from a lone-pair at carbon and an N–I antibonding interaction (Figure 3B). A strong nondynamic electron correlation leads to fractional electron occupation numbers of 1.50 and 0.54 of the σ and σ* orbital, respectively, in a complete active space (CASSCF) treatment. In agreement with this electronic structure, the UV/vis spectrum obtained after λ = 254 nm irradiation of an argon matrix containing **2** showed a broad feature centered at 416 nm, that can be assigned to a σ–σ* transition with the aid of time-dependent density functional theory computations (Figure S2). Furthermore, the 416 nm band got bleached on exposure of the matrix to room light or upon irradiation with λ = 290–320 nm (Figure S3) as expected from the preceding IR experiments.

**Figure 3:**
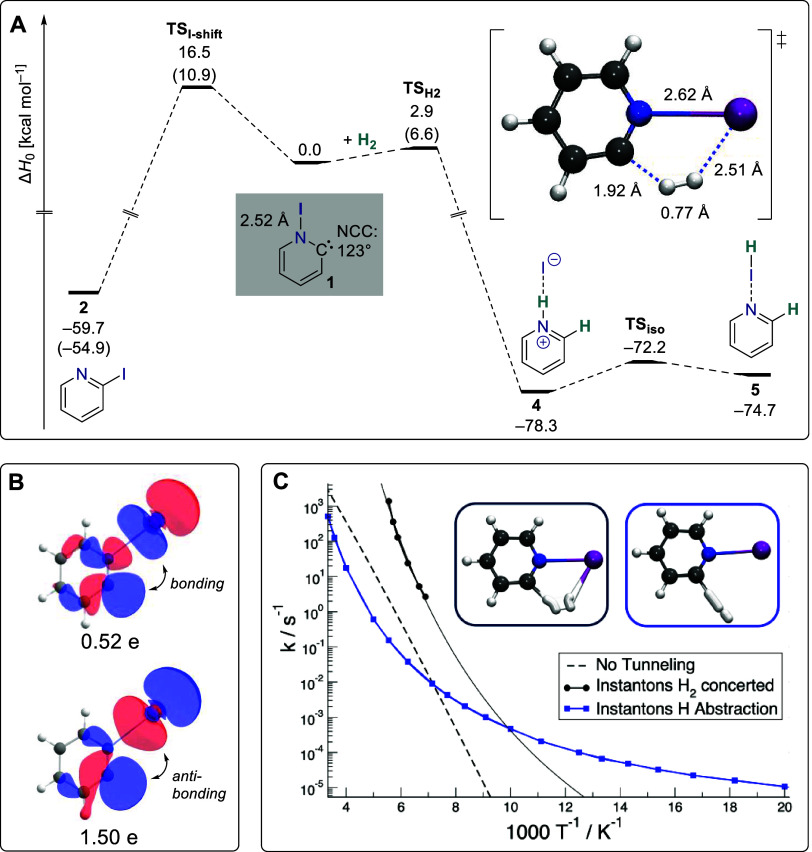
Stability, electronic structure, and hydrogenation of **1**. (A) Potential energy surface; normal: DLPNO–CCSD­(T)/def2-QZVPP//B2PLYP-D3/def2-TZVPP; parentheses: (NEVPT2/def2-TZVPP//CAS­[(12,11)/(10,9)]/def2-TZVPP). (B) Selected CASSCF orbitals and their electron occupation numbers. (C) Arrhenius plot of the H_2_ activation mechanisms. At cryogenic temperatures, the concerted H_2_ activation mechanism (black) switches to a hydrogen atom abstraction mechanism (blue).

To gain further insight into the electronic structure of **1**, we replaced the iodine substituent at the nitrogen atom with increasingly strongly bound groups (Br, Cl, F, CH_3_; Figure 4). The computed bond dissociation energies (**BDEs**), when the N–X bond is cleaved into the corresponding radicals, ranges from approximately 10 kcal mol^–1^ in the case of iodine to almost 50 kcal mol^–1^ for the methylated Hammick intermediate. A strong correlation of the electrons in the σ-type frontier orbitals, which might be understood as a σ-resonance stabilization, can only be relevant when the σ*_N–X_ orbital is energetically available, i.e., when the N–X bond is weak. Accordingly, there is a transition in the electronic structure from σ/σ* to σ/π frontier orbitals in between the chlorine and fluorine substituents, that comes along with an increase in BDE from 18.0 kcal mol^–1^ to nearly 35 kcal mol^–1^ (Figures 4, S11). The electronic reorganization is reflected structurally in the carbene bond angles which are larger for σσ*-carbenes (>120°), to facilitate σ orbital overlap, than for the ordinary σπ-carbenes (112°). Despite their weak N–X bonds (Figures 4, S11), σσ*-carbenes enjoy a significant electronic stabilization featuring carbene stabilization energies (CSEs) of around 80 kcal mol^–1^ akin to their σπ-congeners (Figure 4); the CSE measures carbene stability based on hydrogenation energies in comparison to methylene.^28^ In addition, all considered σσ*-carbenes prefer the singlet over the triplet as the ground state (cf. Δ*E*_ST_, Figure 4), but will inherently display singlet diradical character due to their electronic structure.^29^

**Figure 4:**
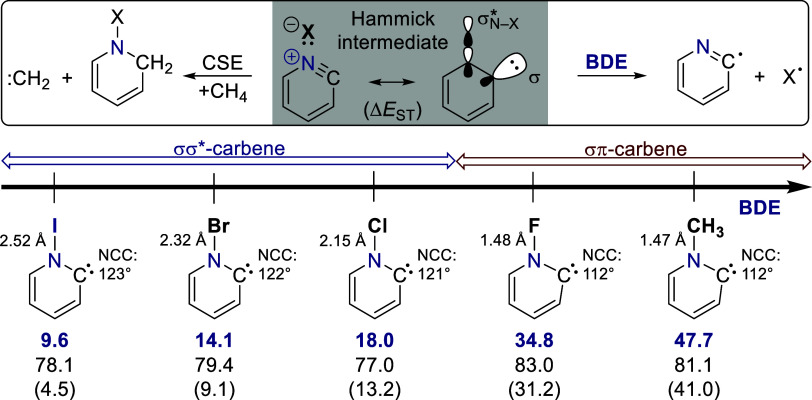
*N*-substitution dependent electronic structure of Hammick intermediates. Dependence of the electronic structure of pyridinylidenes depending on the bond dissociation energies (blue, bold) of different substituents (I, Br, Cl, F, CH_3_) at the nitrogen atom into the corresponding radicals at the DLPNO–CCSD­(T)/def2-QZVPP//B2PLYP-D3/def2-TZVPP level of theory in units of kcal mol^–1^. The carbene stabilization energy (normal) and the singlet–triplet gap (in parentheses) are evaluated at the same level.

### 2.2 Tunneling-Mediated Hydrogen Activation

Given that the σ and σ* frontier orbitals possess an ideal symmetry for the in-plane interaction with the bonding and antibonding σ orbitals of molecular hydrogen (cf. Figure 3B), respectively, we decided to probe the reactivity of **1** toward this diatomic. Indeed, our computations suggest that the reaction proceeds with a barrier of merely 2.9 kcal mol^–1^ (6.6 kcal mol^–1^) at the DLPNO–CCSD­(T) (NEVPT2) level of theory leading to the production of pyridinium iodide, **4** (Figure 3A). This finding is in stark contrast to the ordinarily observed geminal dihydrogenation reactivity of Hammick intermediates and all other carbenes.^20,30–35^

For the experimental validation of these predictions, **2** was codeposited with a large excess of neon gas doped with 3% of H_2_ applying conditions otherwise identical to the previous experiments. The UV irradiation (λ = 254 nm) of this matrix again led to the bleaching of **2** within minutes and the observation of new IR bands as shown in the difference IR spectrum of Figure 5A (see Figure S4 for the extended spectrum), which are markedly different from the experiment without hydrogen. Although pyridinium iodide is the anticipated product from intrinsic reaction path (IRC) computations, only a subset of the evolving bands at 1199, 1160, 979, 748, 687, and 602 cm^–1^ show a convincing agreement with the computed IR spectrum of the ion pair **4** (Figure 5D). As expected from the presence of an ionic hydrogen bond, bands associated with intermolecular motion are strongly broadened like the feature at 979 cm^–1^ and a greatly extended absorption in the range of 1400–2200 cm^–1^ corresponding to the parallel stretching vibration of the intermolecular proton bond (Figures 5A, S4).^36,37^

**Figure 5:**
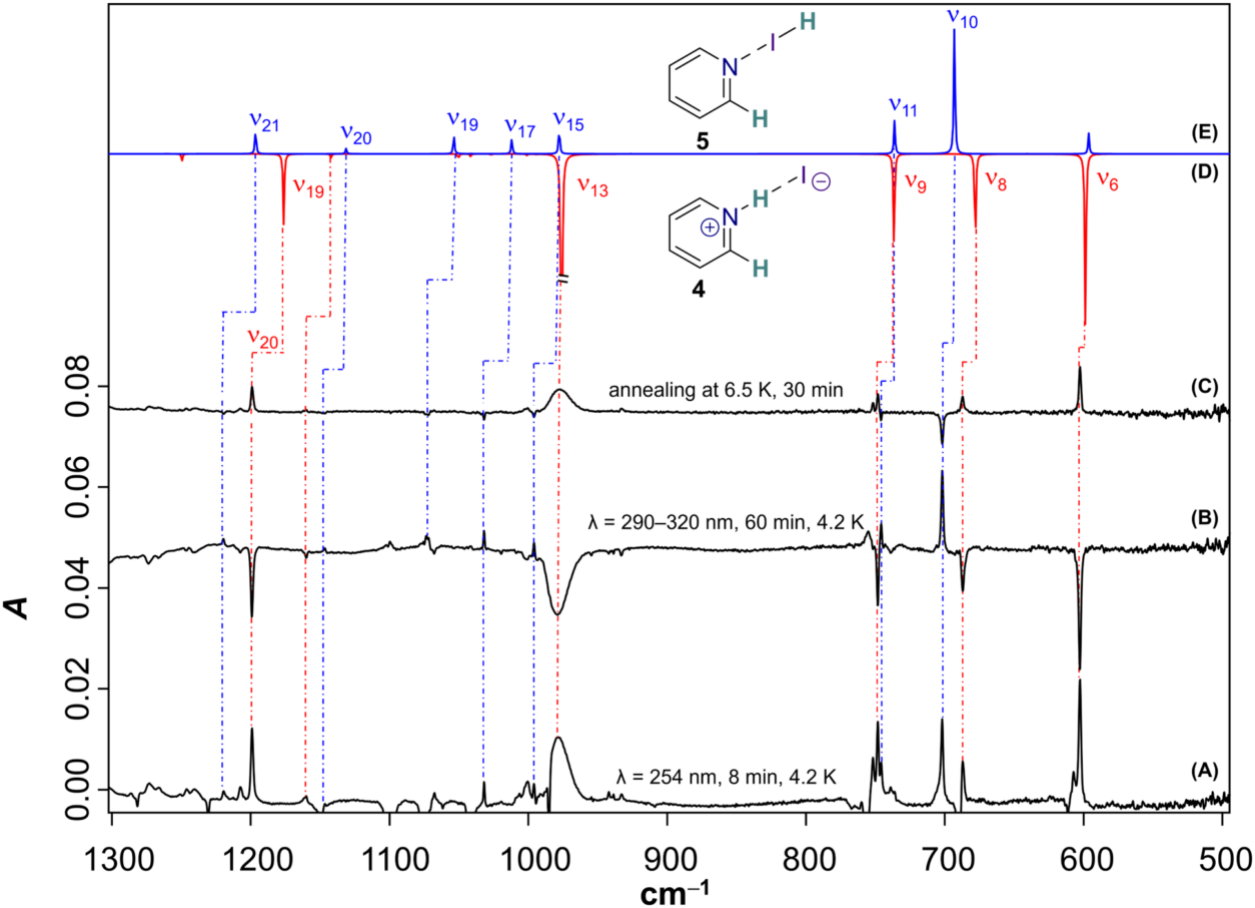
Reaction of **1** with H_2_. (A) Difference IR spectrum after irradiation of a neon matrix, doped with 3% H_2_, and containing **2**, for 8 min with λ = 254 nm. (B) Difference IR spectrum after irradiating this matrix for 60 min with λ = 290–320 nm. (C) Difference IR spectrum after annealing the doubly irradiated matrix for 30 min at 6.5 K. (D) and (E) Computed harmonic spectra (scaled, scaling factor (SF): 0.9605) of **4** and **5**, respectively (B2PLYP-D3/def2-TZVPP).

To aid the identification of additional species, the matrix was subjected to secondary irradiation with λ = 290–320 nm. The resulting difference IR spectrum, given in Figure 5B, showed bleaching of the subset of IR bands assigned to **4** and a concomitant increase of all remaining, so far unassigned IR bands located at 1220, 1147, 1073, 1032, 996, 746, and 702 cm^–1^. Thus, we envisioned that the additional product might be an isomer of **4** with a less strong intermolecular interaction. Surely, we were able to locate the halogen-bound complex of hydrogen iodide with pyridine, **5**, that is only slightly less stable than **4** by 3.6 kcal mol^–1^ (Figure 3A). The computed IR spectrum of **5** (Figure 5E) is in convincing agreement with the photoproduct of the secondary irradiation (Figure 5B, λ = 290–320 nm). Moreover, an IR band observed at 2182 cm^–1^ can be identified with the I–H stretching vibration (Figure S4). A back-reaction of the halogen-bound complex **5** to ion pair **4** can be initiated by allowing the matrix to warm to 6.5 K for 30 min, which becomes evident from the difference IR spectrum in Figure 5 **C**. Accordingly, the back-reaction from **5** to **4** is associated with only a minor barrier (Figures 3, S5) and might benefit from QMT contributions at the low temperature of the experiment. Based on these observations, we assign the formation of two different products, **4** and **5**, in the hydrogenation reaction of **1** that was observed under conditions of continuous irradiation (λ = 254 nm).

At this point, it remains unclear whether the hydrogenation reaction proceeds photochemically, in a hot ground state after photochemical formation of **1**, or by QMT. Luckily, we were able to observe a minor amount of **1** in an additional hydrogenation experiment after the initial UV irradiation, which slowly got depleted upon annealing at 6.5 K over hours (Figure S5). Thus, it can be concluded that **1** is a reactive species in the observed hydrogenation reaction, which also proceeds in the absence of light. The lower rate of the dark process indicates that the reaction benefits from residual excess energy after the photochemical formation of **1** and may consequently involve vibrationally activated quantum mechanical tunneling.^38^ Further insights came from utilization of D_2_ instead of H_2_ with reaction conditions otherwise identical. Here, UV irradiation (λ = 254 nm) only results in the production of **1** and no reaction was observed upon further annealing at 7 K for 18 h or on irradiation with different wavelengths (Figure S7). These observations strongly point toward a major kinetic isotope effect and, therefore, indicate that QMT is the driving factor of the reaction.

To further support our experimental findings, we computed tunneling rate constants with instanton theory; an instanton corresponds to the most probable tunneling pathway of a given temperature. We optimized the instantons at the (12,11)-CASSCF/cc-pVDZ-pp level of theory and corrected the potential energy by computing (12,11)-NEVPT2/def2-TZVPP single point energies. At this point, we note that, while the qualitative conclusions remain valid, the calculated tunneling rates carry considerable uncertainty due to their exponential sensitivity to the barrier height. This is reflected in the, albeit small, energy difference between the DLPNO–CCSD­(T) and NEVPT2­(12,11) methods (2.9 *vs.* 6.6 kcal mol^–1^). Surprisingly, instantons for the concerted hydrogen fission reaction could only be optimized down to a temperature of 145 K (Figure 3C, black). Yet, an alternative tunneling mechanism became available, which is based on a hydrogen atom abstraction reaction.^24^ In this tunneling mechanism, the proximal hydrogen atom is transferred to the carbene’s carbon atom while the other hydrogen atom remains unbound (Figure 3C, blue). The latter hydrogen atom can later be trapped by the iodine atom without barrier. For this abstraction mechanism, instantons could be optimized from 320 K down to 50 K. Curiously, all attempts to optimize a saddle point associated with this hydrogen atom abstraction mechanism remained unsuccessful using the (12,11)-CASSCF/cc-pVDZ-pp level of theory suggesting the nonexistence of this stationary point. Thus, the alternative abstraction mechanism only becomes possible through QMT and is favored over the thermal concerted pathway despite a higher energy barrier by a *tunneling control* ^39^ of this chemical reaction. The rate constants of the two mechanisms cross at around 100 K, rendering the hydrogen atom abstraction the predominant mechanisms of H_2_ activation at low temperatures.^40^ At 50 K, the reaction rate constant computed with dual-level instanton theory amounts to 1.07 × 10^–5^ s^–1^, corresponding to a half-life of around 18 h, which is on the order of the experimental values.

## 3 Conclusions

In conclusion, we have discovered the photochemical formation of the *N*-iodo Hammick intermediate representing the first example of a so far unexplored class of carbenes, in which the lowest unoccupied molecular orbital is not a π but a σ* orbital. This introduces a new reactivity into carbenes like the facile sideways addition of molecular hydrogen by QMT. Utilization of such reactive intermediates in synthetic organic chemistry will facilitate new transformations in carbene chemistry like the *syn*-addition to double bonds under cleavage of the substituent on the nitrogen atom.

## 4 Methods

### 4.1 Matrix Isolation Experiments

Two matrix isolation setups were used to carry out experiments under cryogenic conditions. A SHI CKW-21A displex closed-cycle helium cryostat, which reaches a lowest temperature of 4.4 K, was used for the infrared (IR) experiments, while an APD HC-2 displex closed-cycle helium cryostat with a lowest temperature of 6 K was employed in the ultraviolet and visible (UV/vis) experiments. In addition, low pressures of around 10^–6^ mbar were maintained throughout the experiment using an Edwards’ oil diffusion pump. The matrix host gas (neon or argon, Messer-Griesheim, 99.9999%) doped with 3% of H_2_ (Westfalen, 99.999%) or D_2_ (Sigma-Aldrich, 99.96 atom % D) was codeposited with the precursor 2-iodopyridine **2** (Fluorochem, 97%), which was immersed in a cold bath kept at temperatures between −20 to −15 °C, onto a cold CsI window. In order to maintain a constant flow rate of the host matrix gas, an MKS mass flow PR400B controller was used. Furthermore, for annealing the matrix up to a certain temperature, a Lakeshore temperature controller (model 332), was utilized. To induce the described photochemical transformations, a low-pressure mercury lamp (λ = 254 nm, PenRay) and a high-pressure mercury lamp (USHIO, USH-508S) were brought to use. The matrix isolation experiments were analyzed using infrared and ultraviolet and visible UV/vis spectroscopy. For IR spectroscopic measurements, a Bruker Vertex 70 spectrophotometer with a standard resolution of 0.5 cm^–1^ was used to record the IR bands in the region of 4000–400 cm^–1^. UV/vis measurements were made using the PerkinElmer 1050 spectrophotometer.

### 4.2 Computational Details

The geometry optimizations of different species were performed by utilization of the B2PLYP functional^41^ in combination with Grimme’s D3 correction^42^ and the Ahlrichs’ def2-TZVPP basis set.^43^ To characterize the optimized geometries as minima or first-order saddle points and to obtain zero-point corrections to electronic energies, vibrational frequency computations were carried out employing the harmonic approximation. Moreover, the vibrational frequencies were used to obtain the calculated IR spectrum of different chemical species, which were scaled with a recommended scaling factor.^44^ The anharmonically corrected vibrational frequencies and IR spectra were computed with second-order vibrational perturbation theory (VPT2).^45^ The DFT energies of the stationary points were refined using domain based local pair natural orbital (DLPNO) coupled cluster theory with single, double, and a perturbative estimate of triple excitations [DLPNO–CCSD­(T)],^46–48^ combined with def2-QZVPP basis set.^43^ In addition, the geometry optimization of differently substituted Hammick intermediates was performed utilizing the complete active space self-consistent field (CASSCF)^49,50^ theory and the def2-TZVPP basis set. An active space of 10 electrons and 9 orbitals, as shown in Figure S12, was used in the CASSCF calculations. Dynamical correlation energy corrections to the CASSCF/def2-TZVPP energies at the optimized geometries were added by performing single point calculations using n-electron valence state perturbation (NEVPT2) theory without the frozencore approximation,^51^ which was combined with the def2-TZVPP basis set.

### 4.2.1 Instanton Theory Computations

To compute instantons, the complete active space self-consistent field (CASSCF)^50,52,53^ method was used in combination with the cc-pVDZ-pp basis set^54,55^ (with the ECP28MDF effective core potential for iodine) in Molpro version 2022.3.^56,57^ For the optimization of the instantons and Hessians built from gradients, the DL-Find program was used.^58^ The tcl-version of ChemShell^59^ was used to interface DL-Find and Molpro. Instantons were optimized using a quadratically converging quasi-Newton–Raphson optimizer^60^ until the maximal component of the gradient does not change by more than 10^–8^ atomic units. 77 images were used to optimize the tunneling path (which represents one of the two identical halves of an instanton).

Instantons could be optimized for the concerted H_2_ activation mechanism for temperatures of 180 to 145 K (crossover temperature *T*_C_ = 175.9 K) and for the H atom abstraction mechanism for temperatures of 320 to 50 K (no saddle point localizable). To improve the accuracy of the instanton rate constants, we used a dual-level approach to correct the potential energy term of the instanton rate constant of these two processes by performing single-point energy calculations.^61^ For this, an electronic structure method of higher accuracy was used without the need of computing the Hessians with the more expensive electronic structure method. In this work, we used the NEVPT2 method in combination with the def2-TZVPP^43,55^ basis set implemented in Orca Version 5.0.1.^62^

## Supplementary Material



## Notes

IR and UV/vis spectra, computational data, band positions and intensities, Cartesian coordinates and energies, instanton theory details (PDF)
