# Supplementary material for: Hydrogen Activation by a σσ*-Carbene Through Quantum Tunneling
Source: J Am Chem Soc. 2025 Jul 15;147(39):35275–82. doi: 10.1021/jacs.5c06016 (PMC12498415; doi:10.1021/jacs.5c06016)
Supplement: Supplementary file 1 [file ja5c06016_si_001.pdf]

Supporting Information for

Hydrogen Activation by a σσ*-Carbene Through Quantum Tunneling

Virinder Bhagat, Jan Meisner, and J. Philipp Wagner

Corresponding authors: philipp.wagner@orgchem.uni-tuebingen.de, meisner@hhu.de

Table of Contents

1. Additional IR and UV/vis spectra 3
2. Additional computational data 10
3. IR spectroscopy data 13
4. Cartesian coordinates and energies 19
5. Instanton theory computations 25
6. References 27

1. Additional IR and UV/vis spectra

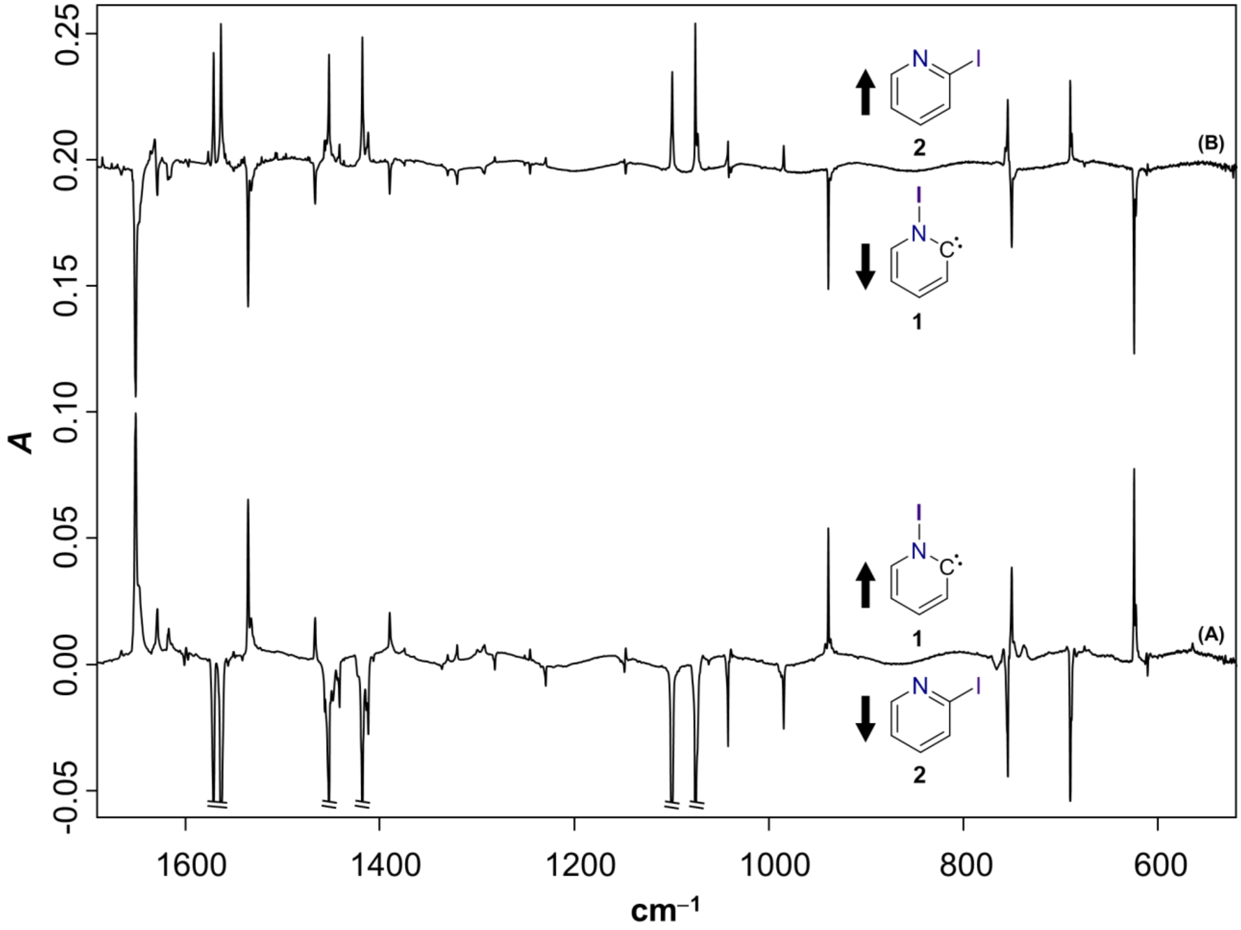

Fig. S1.

(A) Difference spectrum after irradiation of the neon matrix containing **2** for 10 min with λ = 254 nm. **(B)** Difference spectrum after exposing the matrix to the room light for 60 min.

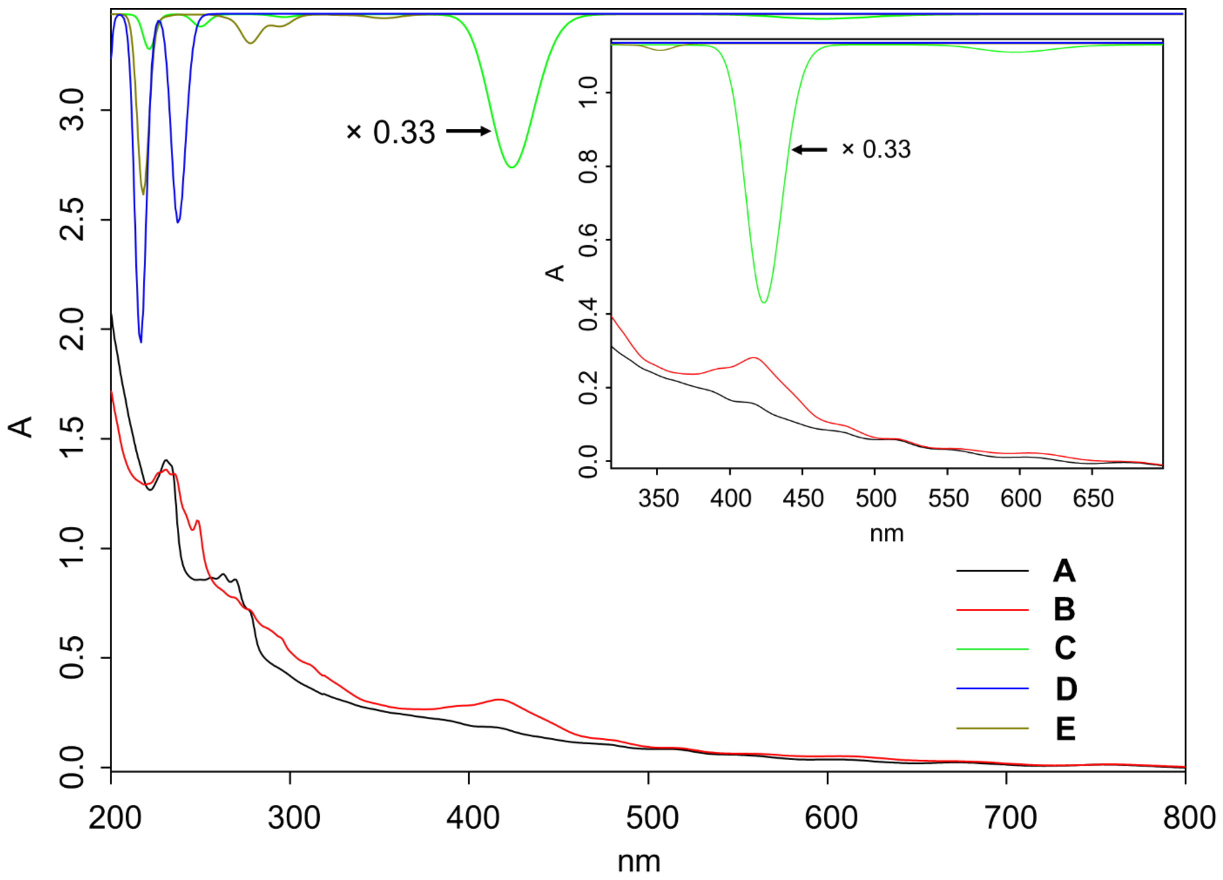

Fig. S2.

A) UV/vis spectrum after co-deposition of precursor **2** with a large excess of argon for 15 min. **B)** Irradiation of the matrix for 5 min with λ = 254 nm. **C)**, **D)**, and **E)** Computed UV/vis spectra of **1**, **2** and **3**, respectively, at the CAM-B3LYP/def2-TZVPP//B2PLYPD3/def2-TZVPP level of theory.

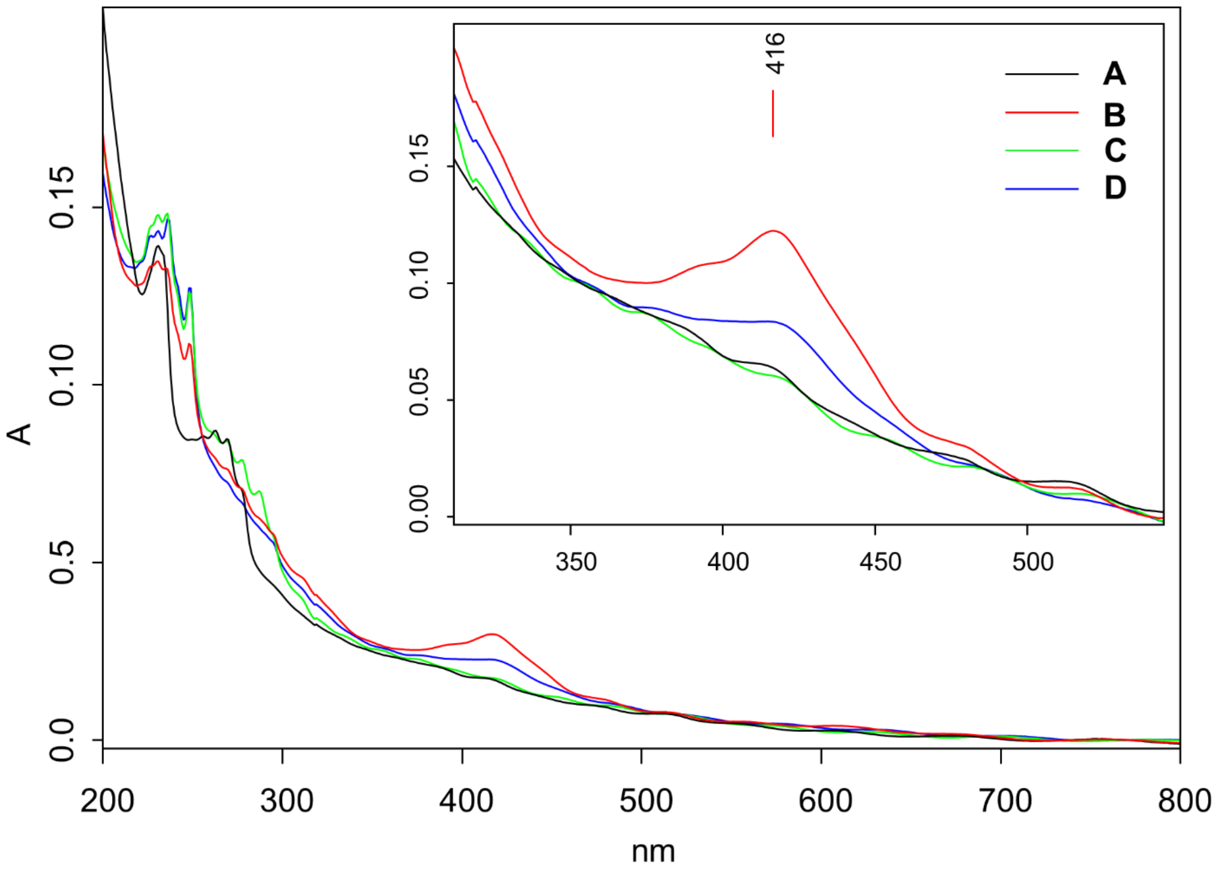

Fig. S3.

A) UV/vis spectrum after co-deposition of precursor **2** with a large excess of argon for 15 min. **B)** Irradiation of the matrix for 5 min with λ = 254 nm. **C)** Irradiation of the matrix for 10 min with λ = 290–320 nm. **D)** Irradiation of the matrix for 5 min with λ = 254 nm.

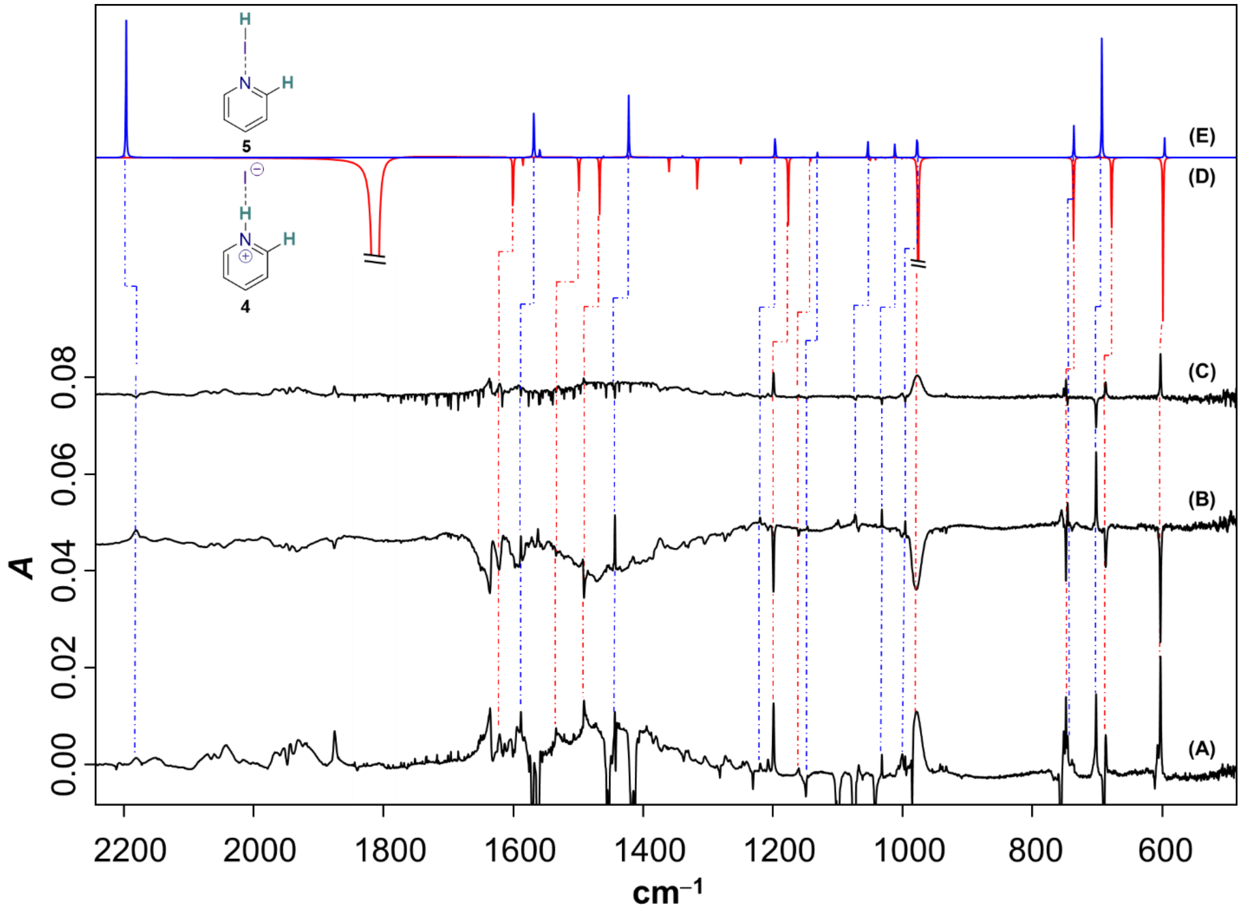

Fig. S4.

(A) Difference spectrum after irradiation of the neon matrix, which is doped with 3% H_2 and contains **2**, for 8 min with λ = 254 nm. **(B)** Difference IR spectrum after irradiating this matrix for 60 min with λ = 290–320 nm. **(C)** Difference spectrum after annealing the matrix for 30 min at 6.5 K. **(D)** and **(E)** are computed harmonic IR spectra (scaled, scaling factor: SF: 0.9605) of **4** and **5**, respectively, obtained at the B2PLYP-D3/def2-TZVPP level of theory.

sss

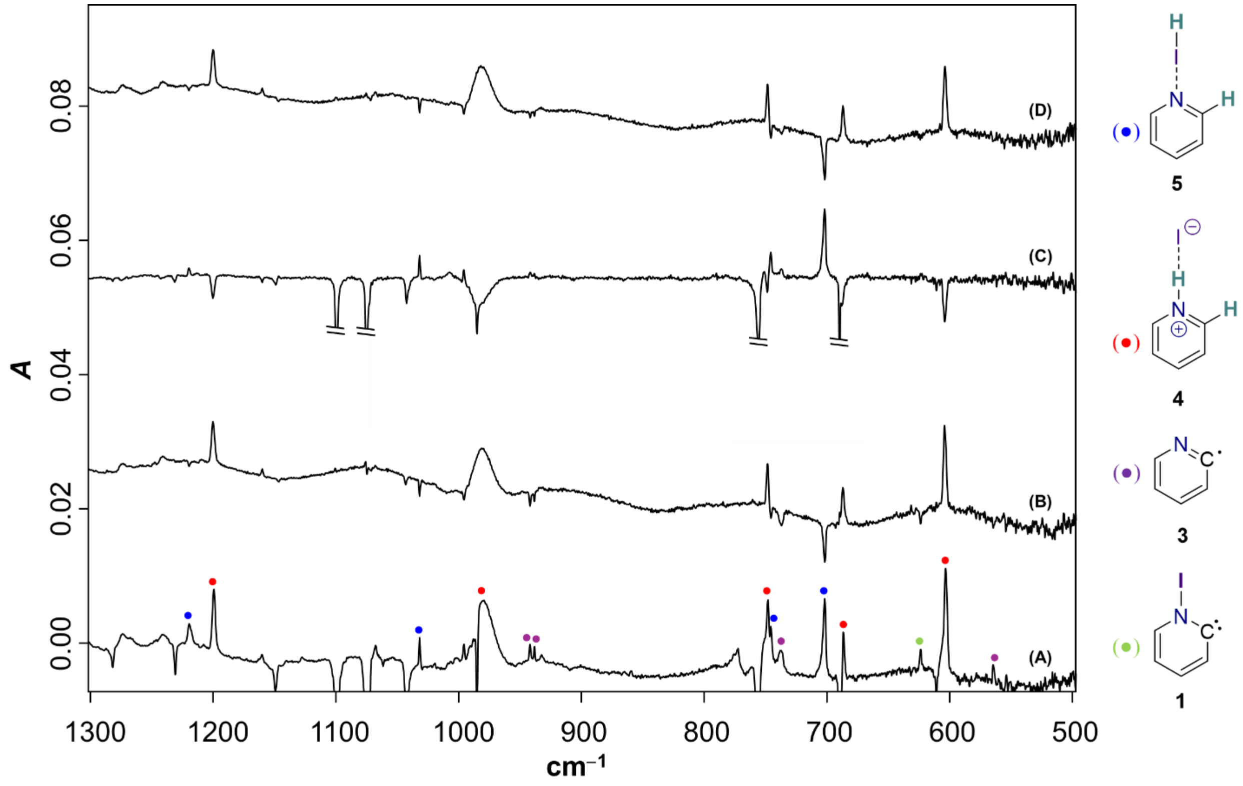

Fig. S5.
A) Difference spectrum after irradiation of a neon matrix, which is doped with 3% H_2 and contains **2**, for 8 min with λ = 254 nm. **B)** Difference spectrum after annealing the matrix in darkness for 14.5 h at 6.5 K. **C)** Difference spectrum after irradiation of the matrix for 6 min with λ = 254 nm. **D)** Difference spectrum after annealing the matrix in darkness for 10 h at 6.5 K.

The difference spectrum **A** was obtained by irradiation of a neon matrix doped with 3 % of H_2 and containing **2**. In this spectrum, four distinct species, **1**, **3**, **4**, and **5**, were identified and their respective IR bands are assigned using the colored symbols shown on the right-hand side of the Figure. Observation of additional species, **1** and **3**, might be due to the deposition of an increased amount of **2** in the experiment compared to the experiment corresponding to Fig. 5. Subsequently, keeping the matrix in the dark over hours at 6.5 K, led to the conversion of **5** to **4** as shown in the difference spectrum **B**. Further irradiation with λ = 254 nm resulted in the consumption of **2** and **4**, leading to the exclusive formation of species **5 (**spectrum **C**). This indicates that species **5** is a secondary product of the photoirradiation. Finally, the observations corresponding to the difference spectrum **D** were similar to that of spectrum **B**.

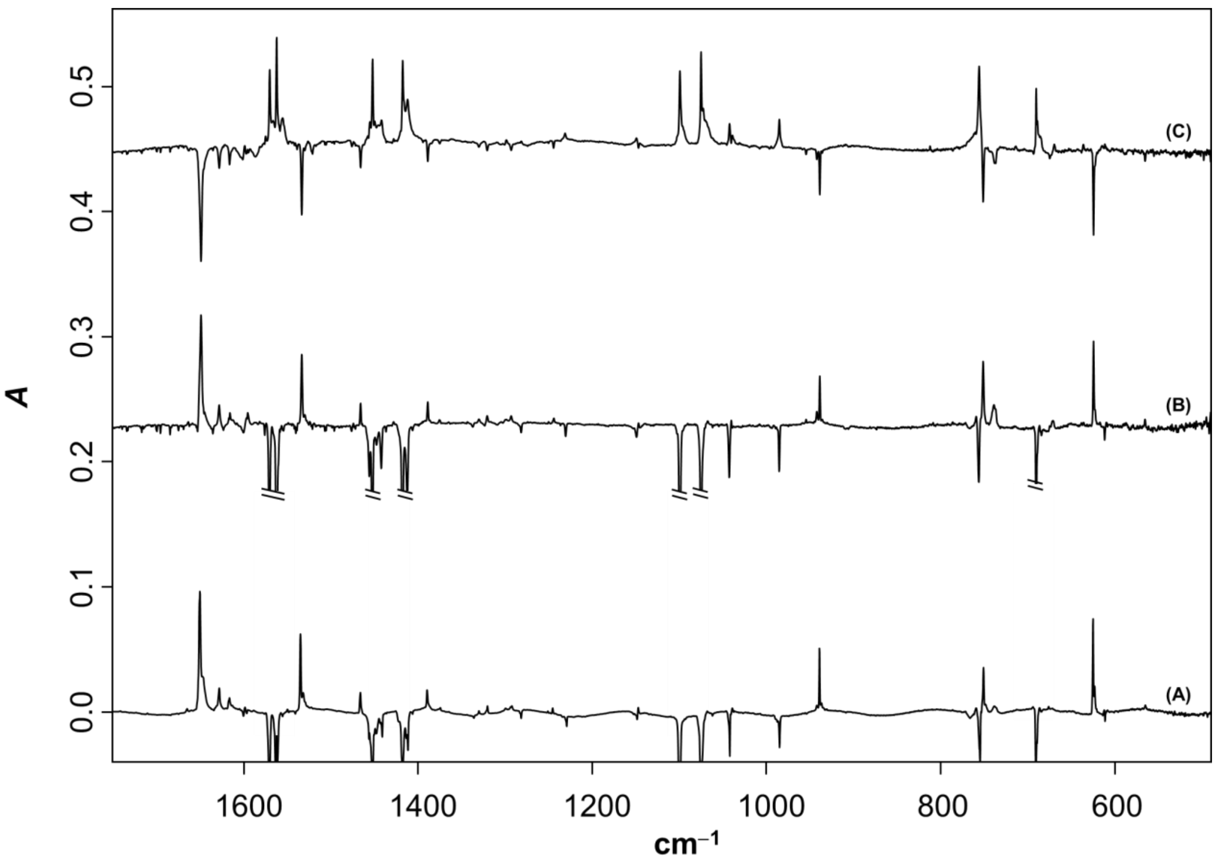

.

Fig. S6.

A) Difference spectrum after irradiation of a neon matrix (without doped H_2 or D_2) containing **2**, for 10 min with λ = 254 nm. **B)** Difference spectrum after irradiation of the neon matrix, which is doped with 3% D_2 and contains **2**, for 7 min with λ = 254 nm. **C)** Difference spectrum after irradiation the D_2-doped neon matrix for 30 min with λ = 290–320 nm.

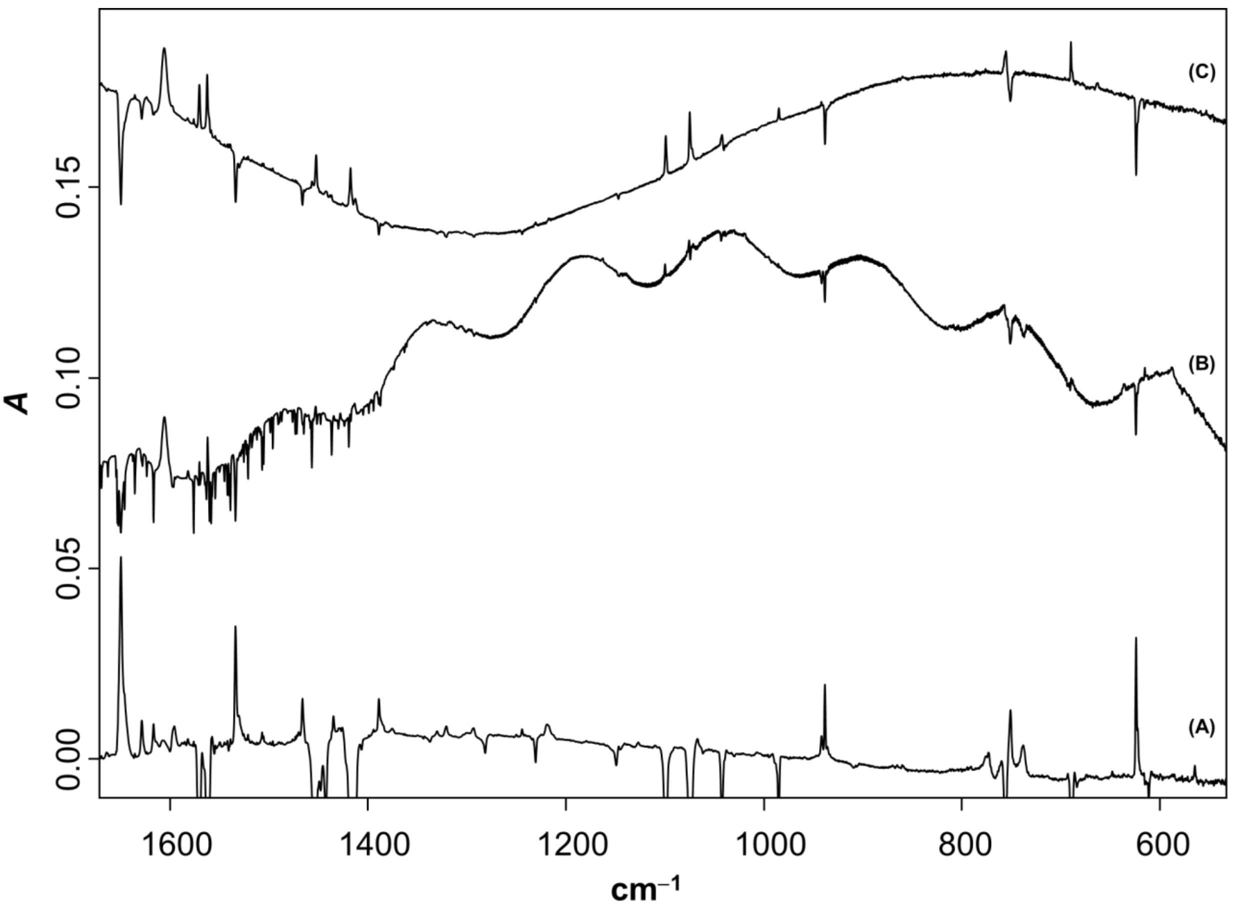

Fig. S7.

A) Difference spectrum after irradiation of a neon matrix, which is doped with 3% D_2 and contains **2**, for 8 min with $\lambda = 254$ nm. **B)** Difference spectrum after annealing the matrix for 18 h at 7 K. **C)** Difference spectrum after exposing the matrix to room light for 60 min.

The difference spectrum **A** was obtained after the irradiation of a neon matrix doped with 3% of D_2 and containing **2**. In this spectrum, a major amount of **1** and a minor amount of **3** could be identified, with no evidence for the formation of a D_2 addition product. Additionally, annealing of the matrix at 7 K did not induce any reaction between **1** and D_2, as shown in spectrum **B**. Furthermore, species **1** was predictably converted back to **2** after exposing the matrix to room light for 60 min.

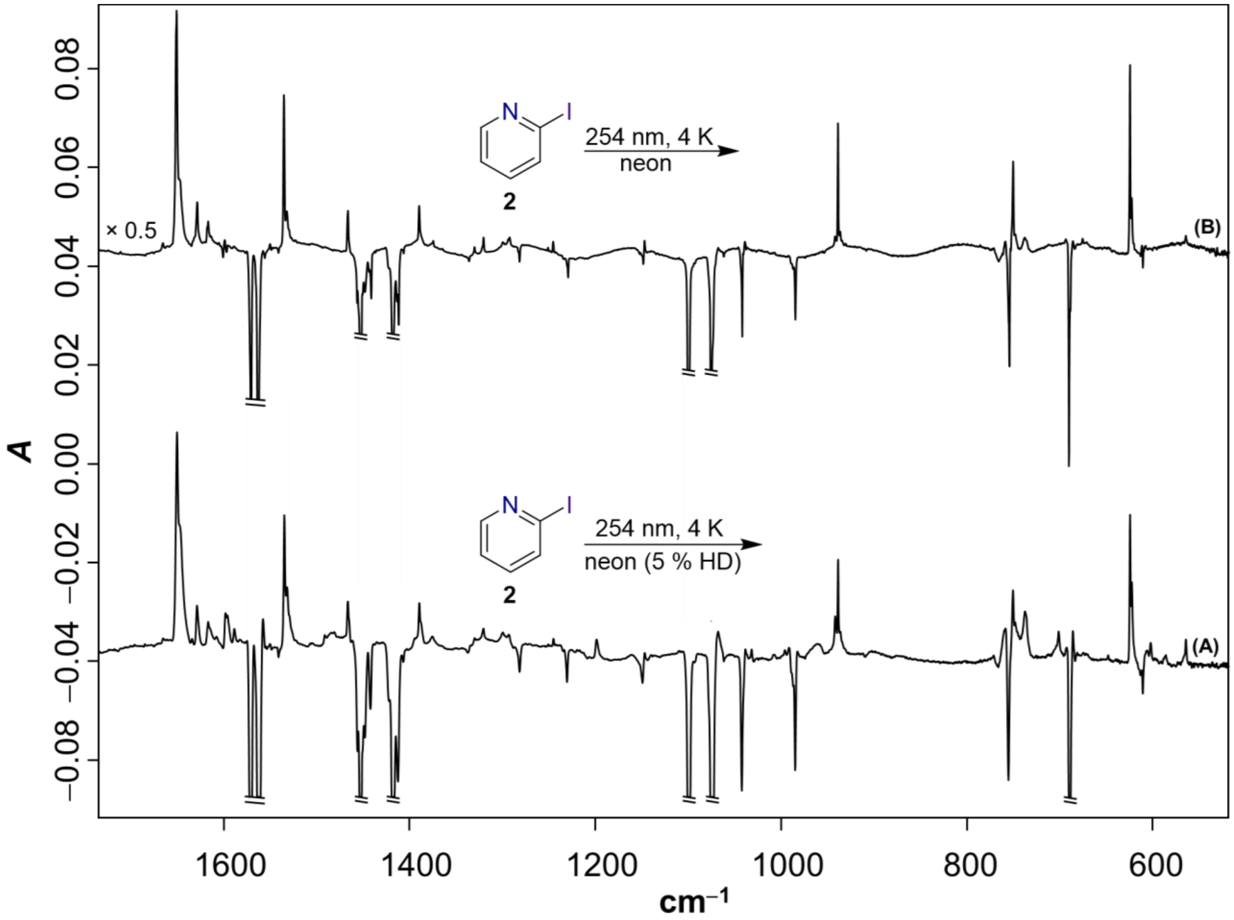

Fig. S8.

A) Difference spectrum after irradiation of a neon matrix, which is doped with 5 % HD and contains **2**, for 8 min with λ = 254 nm. **B)** Difference IR spectrum after irradiation of an undoped neon matrix containing **2** for 10 min with λ = 254 nm.

The spectra reveal that the major product of 2-iodopyridine photolysis is carbene **1** in the presence of HD indicating a slow reaction with partially deuterated hydrogen.

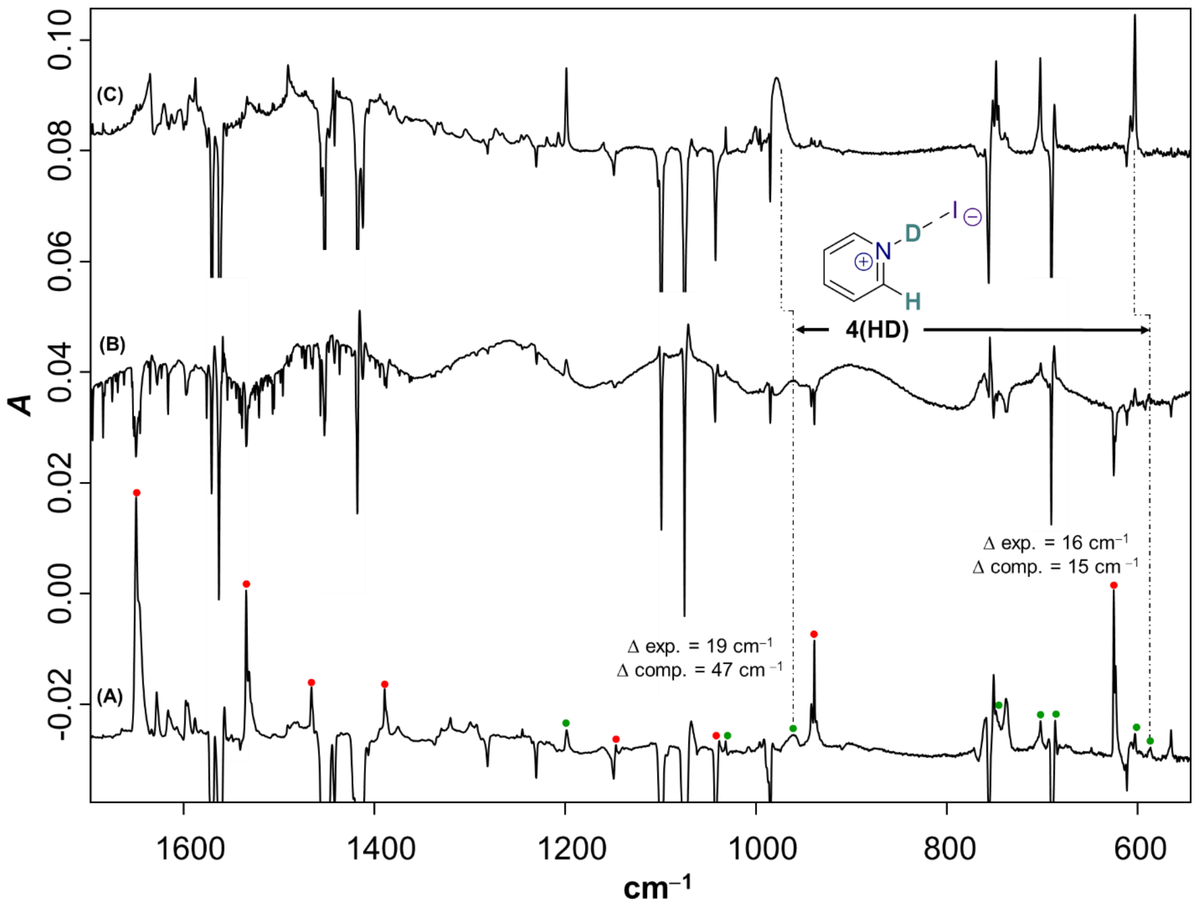

Fig. S9.

A) Difference spectrum after irradiation of a neon matrix, which is doped with 5 % HD and contains **2**, for 8 min with λ = 254 nm. **B)** Difference spectrum after annealing the matrix in darkness for 20 h at 5.5 K. **C)** Difference spectrum after irradiation of the neon matrix, which is doped with 3% H_2 and contains **2**, for 8 min with λ = 254 nm. ● assigned to 2-iodopyridine **1**. ● assigned to H_2/HD addition products.

Spectrum (A) in Fig. S9 contains IR bands (red dots) corresponding to 2-iodopyridine **1** as the major product. However, we could also observe less intense additional IR signals (green dots), the majority of which could be identified as the H_2 addition products **4** and **5,** based on the previous H_2 addition experiments [Fig. 5(A), Fig. S4(A)]. The H_2 addition products, which form within seconds under irradiation conditions, partly result from the presence of a 2% H_2-impurity (standard isotope) in the HD sample used in the experiments. Nevertheless, two IR bands could be exclusively assigned to the HD addition product, **4**(HD), based on their expected red shift, supported by the computed shifts. The increase in the bands exclusive to **4**(HD) is minute, even after 20 hours of annealing of the matrix at 5.5 K, indicating a slow reaction. No evidence was found for the competing isotopomer **4**(DH), in which the deuterium atom is bound to the former carbene's carbon atom. The presence of isotopomer **4**(HD) and the absence for evidence of the competing **4**(DH) is in agreement with the hydrogen atom abstraction reaction mechanism at very low temperatures as predicted by our instanton theory computations.

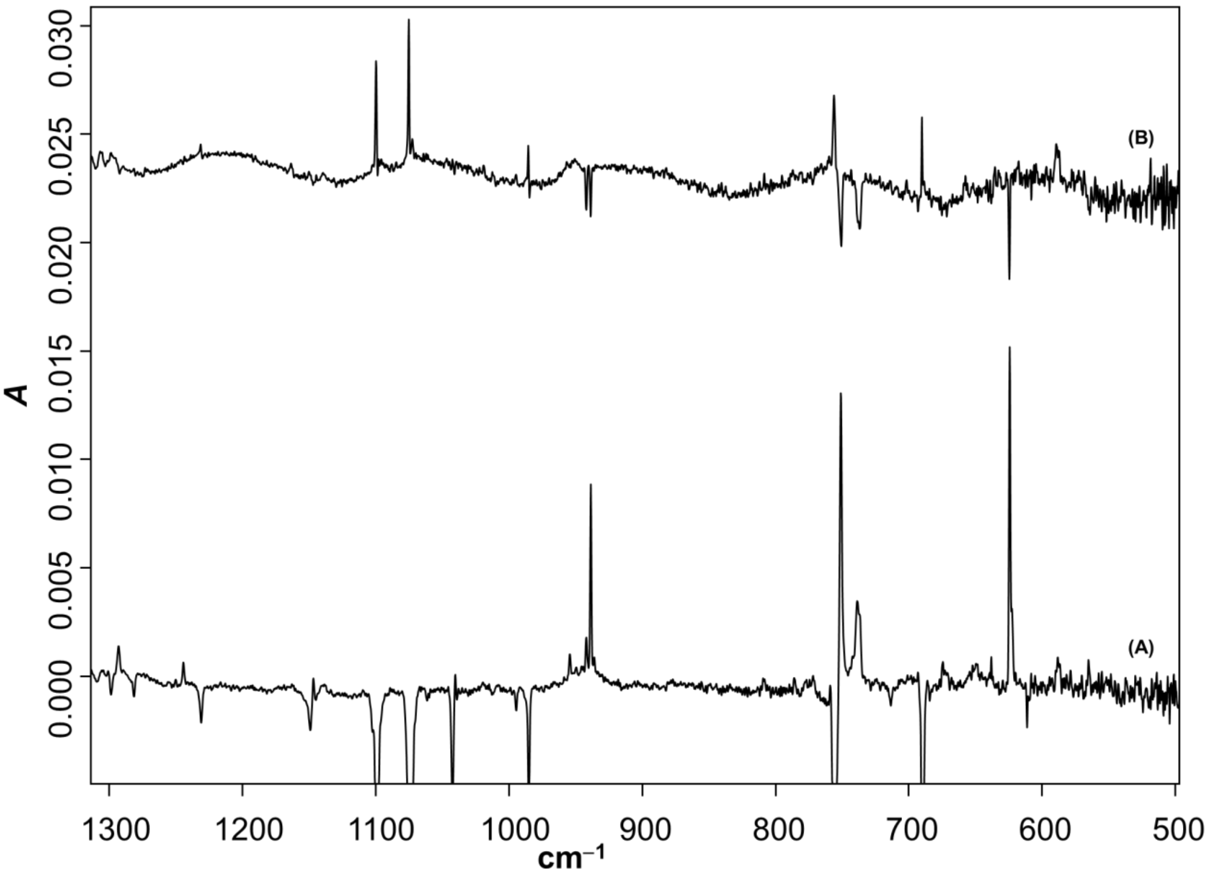

Fig. S10.

A) Difference spectrum after irradiation of a neon matrix, containing **2**, for 5 min with λ = 254 nm. **B)** Difference spectrum after annealing the matrix under dark in darkness 10min at 7.5 K.

The formation of the hydrogenation product could also, in principle, originate from radical **3**, which is present in small amounts and disappears after leaving the matrix in darkness for 14.5 hours at 6.5 K (Fig. S5 B). To test this hypothesis, we performed an additional experiment in which carbene **1** and a minor amount of radical **3** were isolated in an undoped neon matrix (see Fig. S10 above). Upon annealing the matrix to 6.5 K, we observed bleaching of the IR bands associated with radical **3**, accompanied by the formation of precursor **2**. Only a small amount of carbene **1** was bleached, which we attribute to its high light sensitivity. Based on these observations, we conclude that the disappearance of radical **3** in the hydrogenation experiment (Fig. S5 B) results from a radical recombination reaction that regenerates precursor **2**. Thus, the formation of the hydrogenation product most likely originates from carbene **1**.

2. Additional computational data

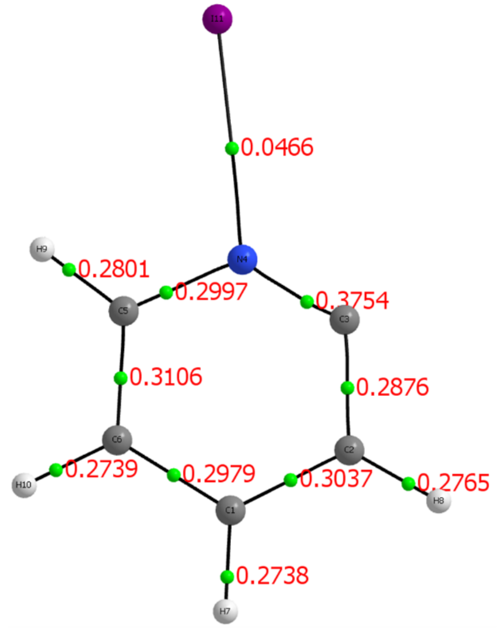

1 (I)

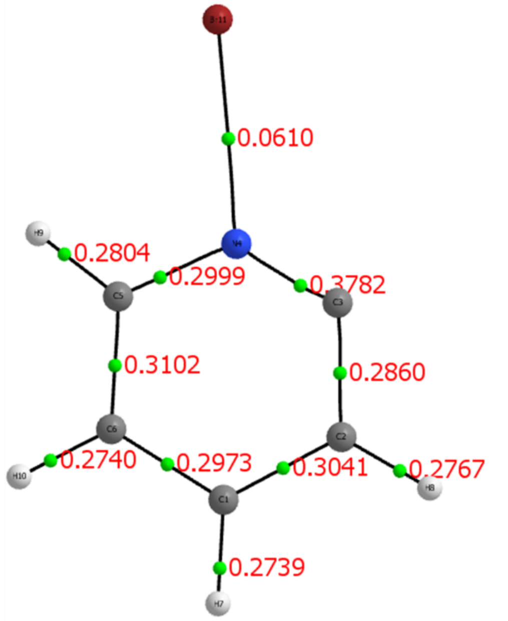

1 (Br)

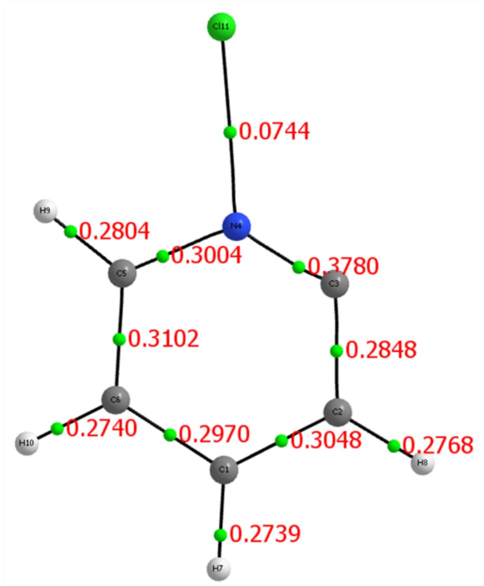

1 (Cl)

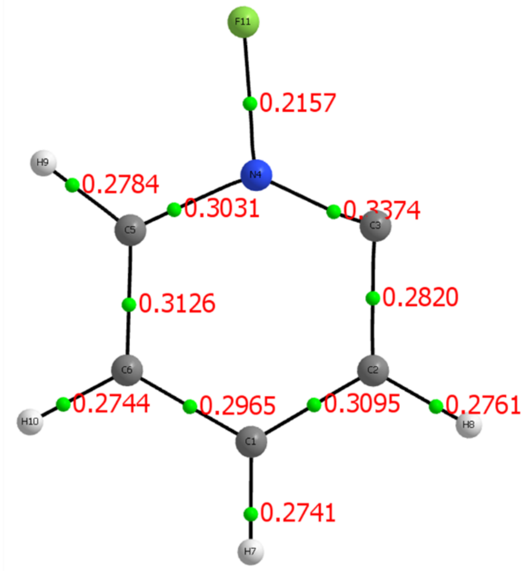

1 (F)

Fig. S11.

Electron densities in atomic units at the bond critical points (BCPs, green balls) of different carbenes computed at their respective optimized geometries at the B2PLYP-D3/def2-TZVPP

level of theory. The electron densities at the BCPs of the N−X bonds are almost an order of magnitude lower than the BCP electron densities at other, “ordinary” bonds.

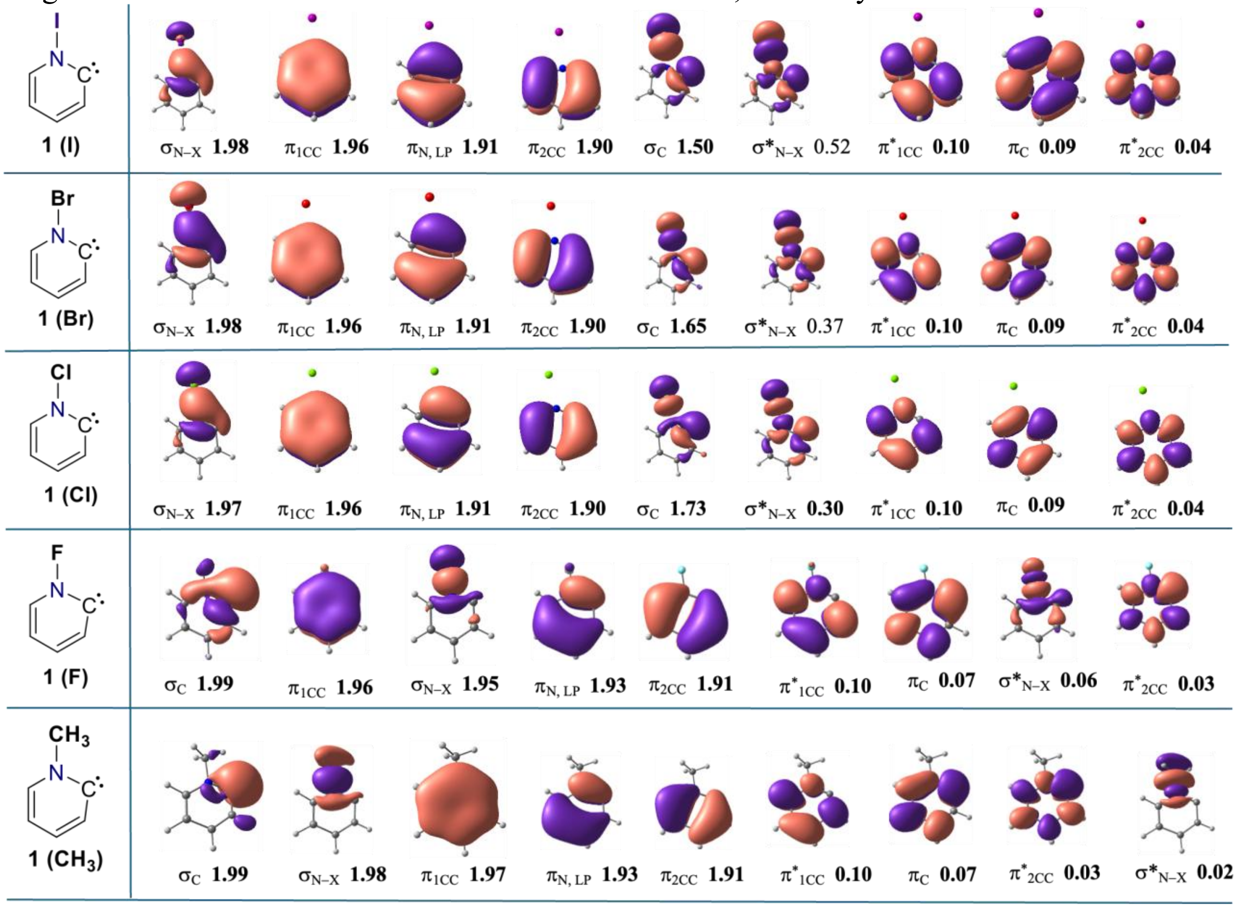

Fig. S12.

Electron occupation of the active space orbitals of different carbenes at their respective optimized geometries calculated at the CASSCF(10e, 9o)/def2-TZVPP level of theory.

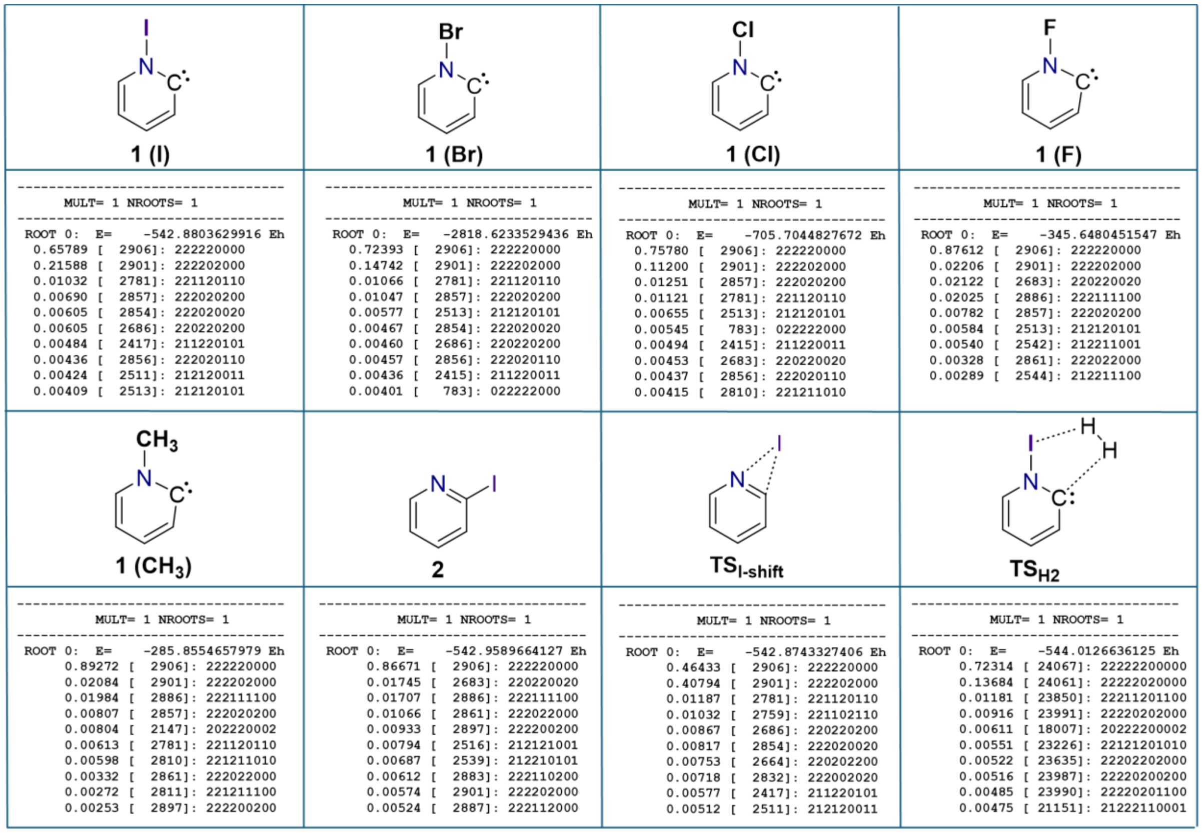

Fig. S13.

Wavefunctions of different carbenes at their respective optimized geometries calculated at the CASSCF[(12e, 11o)/(10e, 9o)]/def2-TZVPP level of theory.

3. IR spectroscopy data

Table S1.
Infrared spectroscopic data of computed (VPT2/B2PLYP-D3/def2-TZVPP level of theory) and observed vibrational frequencies of **1** in the pure neon matrix.

Vibrational Mode, ν^a	Experimental		Computed		approx. description
	ν (cm^{-1})	I^b	ν (cm^{-1})	I^c	
27	-	-	3117	5.5	sym. C-H stretch
26	-	-	3107	1.8	sym. C-H stretch
25	-	-	3089	12.4	asym. C-H stretch
24	-	-	3070	1.4	asym. C-H stretch
23	1651	0.385	1709	100[d]	C-N, C-C stretch
22	1535	0.147	1524	25.6	C-C stretch
21	1466	0.03	1474	10	C-C stretch
20	1390	0.03	1384	4.5	i.p. C-H bend
19	-	-	1352	2	C-C stretch, i.p. C-H bend
18	-	-	1266	1.3	i.p. C-H bend
17	1147	0.006	1161	2	i.p. C-H bend
16	-	-	1107	0.9	i.p. C-H bend
15	1039	0.004	1045	14.9	i.p. C-H bend
14	-	-	1017	4.8	ring breathing
13	-	-	1007	0.1	o.o.p. C-H bend
12	-	-	955	0	o.o.p. C-H bend
11	939	0.073	937	24.5	C-C-C trigonal bend
10	-	-	885	0	o.o.p. C-H bend
9	751	0.07	764	26.9	o.o.p. C-H bend
8	-	-	682	3.1	C-N-C, C-C-C puckering
7	-	-	632	6.7	i.p. ring deformation
6	625	0.126	607	59.6	i.p. ring deformation
5	-	-	450	0	C-N-C, C-C-C o.o.p. bend

[a]Numbering of the vibrational modes is taken from the Gaussian output; [b]obtained after irradiating the neon matrix containing **2** for 10 min; [c]intensity relative to the strongest band; [d]computed absolute intensity: 181.6 km mol^{-1}.

Table S2.

Infrared spectroscopic data of computed (VPT2/B2PLYP-D3/def2-TZVPP level of theory) and observed vibrational frequencies of **1** in the neon matrix doped with 3% of D_2.

Vibrational Mode, ν^a	Experimental		Computed		approx. description
	ν (cm^{-1})	I^b	ν (cm^{-1})	I^c	
27	-	-	3117	5.5	sym. C-H stretch
26	-	-	3107	1.8	sym. C-H stretch
25	-	-	3089	12.4	asym. C-H stretch
24	-	-	3070	1.4	asym. C-H stretch
23	1650	0.105	1709	100^d	C-N, C-C stretch
22	1534	0.039	1524	25.6	C-C stretch
21	1466	0.009	1474	10	C-C stretch
20	1389	0.009	1384	4.5	i.p. C-H bend
19	-	-	1352	2	C-C stretch, i.p. C-H bend
18	-	-	1266	1.3	i.p. C-H bend
17	1147	0.002	1161	2	i.p. C-H bend
16	-	-	1107	0.9	i.p. C-H bend
15	1040	0.003	1045	14.9	i.p. C-H bend
14	-	-	1017	4.8	ring breathing
13	-	-	1007	0.1	o.o.p. C-H bend
12	-	-	955	0	o.o.p. C-H bend
11	939	0.023	937	24.5	C-C-C trigonal bend
10	-	-	885	0	o.o.p. C-H bend
9	751	0.036	764	26.9	o.o.p. C-H bend
8	-	-	682	3.1	C-N-C, C-C-C puckering
7	-	-	632	6.7	i.p. ring deformation
6	624	0.034	607	59.6	i.p. ring deformation
5	-	-	450	0	C-N-C, C-C-C o.o.p. bend

[a]Numbering of the vibrational modes is taken from the Gaussian output; [b]obtained after irradiating the neon matrix containing **2** and doped with 3 % of D_2 for 10 min; [c]intensity relative to the strongest band; [d]computed absolute intensity: 181.6 km mol^{-1}.

Table S3.

Infrared spectroscopic data of computed (harmonic and scaled (scaling factor: 0.9605), B2PLYP-D3/def2-TZVPP) and observed vibrational frequencies of **4** in the neon matrix doped with 3% of H_2.

Vibrational Mode, ν[a]	Experimental		Computed		approx. description
	ν (cm^{-1})	I[b]	ν (cm^{-1})	I[c]	
33	-	-	3117	2.9	sym. C-H stretch
32	-	-	3114	0	asym. C-H stretch
31	-	-	3102	4.8	asym. C-H stretch
30	-	-	3099	0.8	asym. C-H stretch
29	-	-	3091	1.7	asym. C-H stretch
28	1400-2200	-	1813	**3819.8**	N-H, I-H stretch
27	1622	0.025	1601	20.3	C-C stretch
26	-	-	1586	2.7	C-C stretch, N-H bend
25	1534	0.003	1499	11.8	i.p. C-H, N-H bend
24	1491	0.033	1467	16.3	i.p. C-H bend
23	-	-	1361	4.5	i.p. C-H, N-H bend
22	-	-	1317	11.9	C-C stretch, i.p. C-H, N-H bend
21	-	-	1250	1.9	i.p. C-H, N-H bend
20	1199	0.034	1177	19.3	i.p. C-H bend
19	1160	0.005	1143	1.2	i.p. C-H bend
18	-	-	1051	1.3	C-C stretch, i.p. C-H bend
17	-	-	1042	0.7	i.p. C-H, N-H bend
16	-	-	1027	0.2	o.o.p. N-H bend
15	-	-	1002	0.4	ring breathing
14	-	-	995	0.1	o.o.p. C-H bend
13	979*	0.156	977	100[d]	C-N-C, C-C-C puckering, I-H stretch
12	-	-	974	0	o.o.p. C-H bend
11	-	-	917	0	o.o.p. C-H, N-H bend

10	-	-	869	0	o.o.p. C-H bend
9	748	0.032	737	26.2	o.o.p. C-H, N-H bend
8	687	0.015	679	26.1	C-N-C, C-C-C o.o.p. bend
7	-	-	630	0	i.p. ring deformation
6	602	0.073	599	49	i.p. ring deformation, I-H stretch

[a]Numbering of the vibrational modes is taken from the Gaussian output; [b]obtained after irradiating the neon matrix containing **2** and doped with 3 % of H_2 for 8 min; [c]intensity relative to the second strongest band; [d]computed absolute intensity: 155.7 km mol^{-1}.

* This assignment is further supported by a similarly broad band at 904.5 cm^{-1} observed in the pyridine–HCl complex, previously attributed to the antisymmetric stretching vibration of the N–H–Cl unit.[1]

Table S4.

Infrared spectroscopic data of computed (harmonic and scaled (scaling factor: 0.9605), B2PLYP-D3/def2-TZVPP) and observed vibrational frequencies of **5** in the neon matrix doped with 3% of H_2.

Vibrational Mode, ν^a	Experimental		Computed		approx. description
	ν (cm^{-1})	I^b	ν (cm^{-1})	I^c	
33	-	-	3099	10.5	sym. C-H stretch
32	-	-	3092	22.8	asym. C-H stretch
31	-	-	3077	3.6	asym. C-H stretch
30	-	-	3060	0.6	asym. C-H stretch
29	-	-	3058	19.6	asym. C-H stretch
28	2182	0.015	2197	100	I-H stretch
27	1588	0.009	1569	36.2	C-C stretch
26	-	-	1560	6.3	C-C stretch
25	-	-	1461	0.9	i.p. C-H bend
24	1444	0.006	1423	38.1	i.p. C-H bend
23	-	-	1339	1.1	i.p. C-H bend
22	-	-	1261	0	C-C stretch
21	1220	0.004	1197	14.8	i.p. C-H bend
20	1147	0.001	1132	3.2	i.p. C-H bend
19	-	-	1054	10.8	C-C stretch, i.p. C-H bend
18	-	-	1042	0.1	i.p. C-H bend
17	1032	0.008	1012	8.9	i.p. ring deformation
16	-	-	980	0	o.o.p. C-H bend
15	996	0.002	978	15.7	C-N-C, C-C-C puckering
14	-	-	968	0	o.o.p. C-H bend
13	-	-	927	0	o.o.p. C-H bend
12	-	-	868	0	o.o.p. C-H bend
11	746	0.003	737	19.4	C-N-C, C-C-C o.o.p. bend

10	702	0.054	694	78.2	o.o.p. C-H bend
9	-	-	641	0.1	i.p. ring deformation
8	-	-	597	12.1	i.p. ring deformation, N-I stretch
7	-	-	407	2.5	C-N-C, C-C-C, I-H o.o.p. bend

[a]Numbering of the vibrational modes is taken from the Gaussian output; [b]obtained after irradiating the neon matrix containing **2** and doped with 3 % of H_2 for 8 min; [c]intensity relative to the strongest band; [d]computed absolute intensity: 73.3 km mol^{-1}.

4. Cartesian coordinates and energies

Table S5.

Cartesian coordinates of optimized geometries calculated at the B2PLYP-D3/def2-TZVPP level of theory. Energies are given in Hartree units at the B2PLYP-D3/def2-TZVPP (normal) and DLPNO-CCSD(T)/def2-QZVPP//B2PLYP-D3/def2-TZVPP (italics) levels.

**1**			
-544.949547 (*-544.422344)*			
**C**	0.73518000	3.42150600	0.00000000
**C**	-0.61164800	3.08620100	0.00000000
**C**	-0.88911900	1.71628100	0.00000000
**N**	0.00000000	0.84539200	0.00000000
**C**	1.32751700	1.08877900	0.00000000
**C**	1.71064000	2.41384600	0.00000000
**H**	1.03546900	4.46099900	0.00000000
**H**	-1.39010200	3.83255900	0.00000000
**H**	2.00071400	0.24653400	0.00000000
**H**	2.76212300	2.65716600	0.00000000
**I**	-0.34044600	-1.65046600	0.00000000
**$TS_{I\text{-}shift}$**			
-544.925040 (*-544.396074)*			
**C**	2.25415700	1.84019800	0.00000000
**C**	1.20358100	0.93276300	0.00000000
**C**	-0.49333200	2.44632300	0.00000000
**C**	0.44201300	3.46804900	0.00000000
**C**	1.81131000	3.16176700	0.00000000
**H**	3.29566000	1.56520700	0.00000000
**H**	-1.56226500	2.58983000	0.00000000
**H**	0.10891600	4.49508400	0.00000000
**H**	2.53854200	3.96274500	0.00000000
**N**	0.00000000	1.19469600	0.00000000
**I**	-0.67334400	-1.73717600	0.00000000
**2**			
-545.038240 (*-544.517473)*			
**C**	1.23371300	1.23124200	0.00000000
**C**	0.00000000	0.58179500	0.00000000
**C**	-1.16004200	2.53428300	0.00000000
**C**	0.00479500	3.28847300	0.00000000
**C**	1.22428100	2.61873500	0.00000000
**H**	2.15506400	0.67119600	0.00000000
**H**	-2.13130700	3.01070500	0.00000000

**H**	-0.04350400	4.36692600	0.00000000
**H**	2.15590700	3.16641400	0.00000000
**N**	-1.16719300	1.19484000	0.00000000
**I**	-0.03362800	-1.53030700	0.00000000
**TS_{H2}**			
-546.106731 (*-545.581427)*			
**C**	-0.83568100	3.07721200	0.00000000
**C**	-0.95944400	1.69042100	0.00000000
**C**	1.29069700	1.26812700	0.00000000
**C**	1.54613700	2.62687600	0.00000000
**C**	0.47738600	3.53524500	0.00000000
**H**	-1.68257400	3.74471500	0.00000000
**H**	2.04066100	0.49161900	0.00000000
**H**	2.56761200	2.97541900	0.00000000
**H**	0.67645700	4.59861900	0.00000000
**N**	0.00000000	0.89858900	0.00000000
**I**	-0.15222800	-1.72297400	0.00000000
**H**	-2.38051100	0.39830900	0.00000000
**H**	-2.26813400	-0.36847400	0.00000000
**3**			
-247.416214 *(-247.141994)*			
**C**	1.19945900	0.58672000	0.00000000
**C**	1.17035000	-0.80338800	0.00000000
**C**	-0.09903000	-1.36823500	0.00000000
**N**	-1.21410600	-0.73938200	0.00000000
**C**	-1.19813200	0.60936600	0.00000000
**C**	0.00000000	1.30297900	0.00000000
**H**	2.14598500	1.11010200	0.00000000
**H**	2.07073700	-1.39694700	0.00000000
**H**	-2.15327000	1.11520300	0.00000000
**H**	-0.00059700	2.38266700	0.00000000
**4**			
-546.229038 (*-545.710905)*			
**C**	0.00000000	0.00000000	-3.89726800
**C**	0.00000000	1.20439800	-3.19974700
**C**	0.00000000	1.17092900	-1.81697900
**N**	0.00000000	0.00000000	-1.17783700
**C**	0.00000000	-1.17092900	-1.81697900
**C**	0.00000000	-1.20439800	-3.19974700
**H**	0.00000000	0.00000000	-4.97725000
**H**	0.00000000	2.15152700	-3.71553900
**H**	0.00000000	2.04711100	-1.18528700

**H**	0.00000000	-2.04711100	-1.18528700
**H**	0.00000000	-2.15152700	-3.71553900
**H**	0.00000000	0.00000000	-0.05727900
**I**	0.00000000	0.00000000	2.01255400
**TS_{iso}**			
-546.215536 (*-545.701059)*			
**C**	-4.16752200	0.73612000	0.00599700
**C**	-4.20063700	-0.65270700	0.01114100
**C**	-2.99448700	-1.34563100	0.00391000
**N**	-1.79917700	-0.74992200	-0.00761500
**C**	-1.78204100	0.58572000	-0.01246500
**C**	-2.93126600	1.36960300	-0.00611000
**H**	-5.08290000	1.31054000	0.01113900
**H**	-5.13686200	-1.19126600	0.02039800
**H**	-2.98655700	-2.42833500	0.00746700
**H**	-0.80266000	1.04658200	-0.02206700
**H**	-2.85373700	2.44694900	-0.01085100
**I**	2.32906800	-0.00386700	-0.02893800
**H**	2.47208400	0.11129600	1.56610300
**5**			
-546.220840 (*-545.705149)*			
**C**	0.00000000	0.00000000	-3.90507200
**C**	0.00000000	1.19507400	-3.19638100
**C**	0.00000000	1.14335500	-1.80726200
**N**	0.00000000	0.00000000	-1.11727900
**C**	0.00000000	-1.14335500	-1.80726200
**C**	0.00000000	-1.19507400	-3.19638100
**H**	0.00000000	0.00000000	-4.98561000
**H**	0.00000000	2.14808200	-3.70419200
**H**	0.00000000	2.05296300	-1.22054700
**H**	0.00000000	-2.05296300	-1.22054700
**H**	0.00000000	-2.14808200	-3.70419200
**I**	0.00000000	0.00000000	1.93540700
**H**	0.00000000	0.00000000	3.55364300

Table S6.

Cartesian coordinates of optimized geometries calculated at the CAS[(12,11)/(10,9)]/def2-TZVPP level of theory. Energies are given in Hartree units at NEVPT2/def2 TZVPP//CAS[(12,11)/(10,9)]/def2-TZVPP levels of theory if not mentioned otherwise.

**1**			
-544.348374			
**C**	-3.27657300	-1.18359200	0.00000000
**C**	-1.87498300	-1.16501400	0.00000000
**C**	-1.71359600	1.09530800	0.00000000
**C**	-3.08233900	1.21660500	0.00000000
**C**	-3.88093000	0.05530200	0.00000000
**H**	-3.83122800	-2.09845400	0.00000000
**H**	-1.04921900	1.93411100	0.00000000
**H**	-3.53056600	2.18998300	0.00000000
**H**	-4.95074000	0.14005300	0.00000000
**N**	-1.15905400	-0.13634300	0.00000000
**I**	1.60535600	0.00176800	0.00000000
**$TS_{I\text{-}shift}$**			
-544.331068			
**C**	-2.88618200	-1.76198800	-0.00272000
**C**	-1.58346400	-1.25872000	-0.00786800
**C**	-2.21304500	0.90158500	-0.00323500
**C**	-3.54631300	0.54594300	0.00217000
**C**	-3.89172300	-0.81397200	0.00244600
**H**	-3.08472200	-2.81386100	-0.00278600
**H**	-1.90299000	1.92786200	-0.00363400
**H**	-4.30354800	1.30477900	0.00612800
**H**	-4.92310300	-1.11083400	0.00664400
**N**	-1.23869400	-0.02729800	-0.00823800
**I**	2.52331900	-1.27739800	0.01109300
**2**			
-544.435805			
**C**	-2.35437500	-1.63179400	0.00260600
**C**	-1.19291800	-0.86433600	-0.00043300
**C**	-2.33223400	1.08928000	-0.00363800
**C**	-3.55579400	0.43642800	-0.00063600
**C**	-3.56460700	-0.95480800	0.00255800
**H**	-2.30836400	-2.70056800	0.00499900
**H**	-2.27710800	2.16070700	-0.00614800
**H**	-4.46783200	0.99951000	-0.00082200
**H**	-4.48899300	-1.49915900	0.00493100

**N**	-1.16802000	0.44281500	-0.00345300
**I**	0.71467800	-1.87967700	-0.00026600
**TS$_{H2}$**			
-545.494074			
**C**	-3.19445000	-1.27822200	0.00024900
**C**	-1.80599700	-1.11733600	-0.01815800
**C**	-1.84175800	1.13816400	0.02548300
**C**	-3.21913000	1.13376800	0.04670200
**C**	-3.90681300	-0.09522400	0.03385100
**H**	-3.66307600	-2.23980900	-0.01088700
**H**	-1.25702900	2.03519000	0.03358300
**H**	-3.75772100	2.05998800	0.07272000
**H**	-4.97973700	-0.10886000	0.05014800
**N**	-1.18144500	-0.03349700	-0.00712500
**I**	1.87523100	-0.27188700	-0.06040400
**H**	-0.43113100	-2.24318900	-0.06109900
**H**	0.36874300	-2.10534100	-0.07102500
**1 (Br)**			
-2818.536460 [CAS(10,9)/def2-TZVPP]			
**C**	1.07457300	**-1.13069000**	0.00000000
**C**	2.44534800	-1.17521700	0.00000000
**C**	3.18344400	0.03052300	0.00000000
**C**	2.52251000	1.23820100	0.00000000
**C**	1.11953700	1.14832800	0.00000000
**N**	0.48564100	0.08613400	0.00000000
**H**	4.25611700	-0.00195600	0.00000000
**H**	0.44073200	-1.99106000	0.00000000
**H**	2.94591100	-2.12259500	0.00000000
**H**	3.03228400	2.17847700	0.00000000
**Br**	-1.95708200	-0.15346600	0.00000000
**1 (Cl)**			
-705.617381 [CAS(10,9)/def2-TZVPP]			
**C**	-1.65382900	-0.40565300	-0.00000800
**C**	-0.28388700	-0.39416600	-0.00006200
**C**	0.40725800	0.84241000	-0.00025300
**C**	-0.29569300	2.02483200	-0.00038400
**C**	-1.69769300	1.88419800	-0.00032900
**N**	-2.26930600	0.79811500	-0.00016000
**H**	1.48040500	0.84935200	-0.00029400
**H**	-2.26385200	-1.28207500	0.00013300
**H**	0.25448800	-1.32048200	0.00004300
**H**	0.18032300	2.98251700	-0.00053100

**Cl**	-4.50569700	0.49556100	-0.00005300
**1 (F)**			
-345.560422 [CAS(10,9)/def2-TZVPP]			
**C**	-1.67019200	-0.43782300	0.00001600
**C**	-0.31483600	-0.42329200	-0.00005100
**C**	0.34032700	0.83641300	-0.00024100
**C**	-0.40328700	1.98355900	-0.00036300
**C**	-1.84412400	1.98083200	-0.00032700
**N**	-2.29317800	0.77612700	-0.00013400
**H**	1.41299800	0.87418500	-0.00030800
**H**	-2.29270500	-1.30512000	0.00014200
**H**	0.23011900	-1.34485300	0.00002900
**H**	0.08739500	2.93671600	-0.00053400
**F**	-3.70493000	0.60497600	-0.00012600
**1 (CH_3)**			
-285.72786873 [CAS(10,9)/def2-TZVPP]			
**C**	1.028253000	-1.147344000	-0.000125000
**C**	2.383067000	-1.196123000	-0.000250000
**C**	3.089337000	0.030346000	-0.000032000
**C**	2.383791000	1.200667000	0.000348000
**C**	0.946285000	1.269246000	0.000503000
**N**	0.376597000	0.056225000	0.000240000
**H**	4.163441000	0.027225000	-0.000171000
**H**	0.414896000	-2.025596000	-0.000365000
**H**	2.887484000	-2.141156000	-0.000568000
**H**	2.913307000	2.134319000	0.000513000
**C**	-1.117395000	-0.008770000	0.000021000
**H**	-1.455371000	-0.531432000	-0.884170000
**H**	-1.455629000	-0.532311000	0.883593000
**H**	-1.477642000	1.002773000	0.000464000

5. Instanton theory computations

The potential energy barrier of the concerted H_2 activation pathway becomes lower using NEVPT2/def2-TZVPP level of theory, see Figure S11, left. Also, the potential energy barrier of the H atom abstraction mechanism is lower when using NEVPT2/def2-TZVPP level of theory, see Figure S11, right.

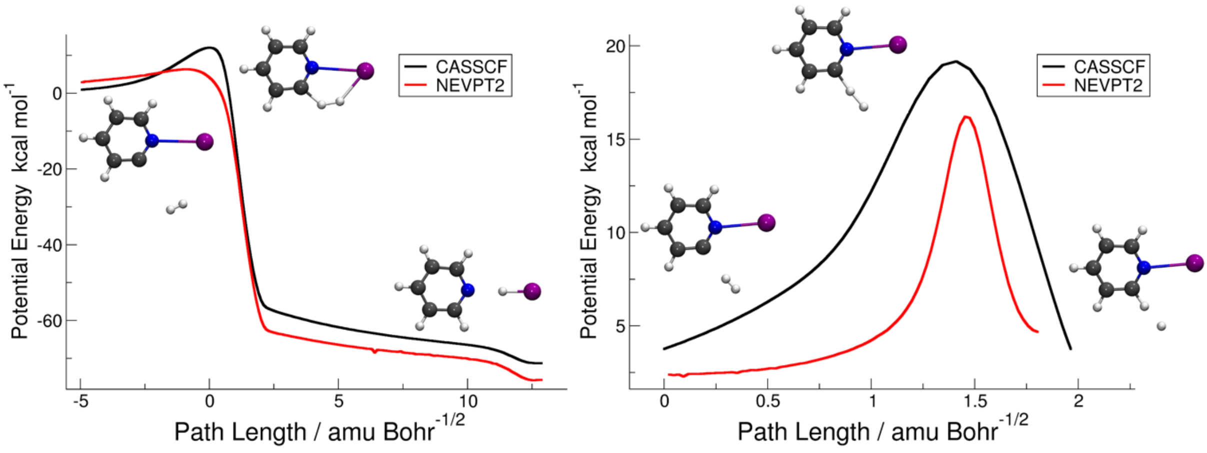

Fig. S11.

Left: Potential energy relative to the energy of the pre-reactive complex along the intrinsic reaction coordinate of the concerted H_2 activation computed using the 12,11-CASSCF/cc-pVDZ-pp level of theory (black line). **Right:** Potential energy relative to the energy of the pre-reactive complex along the instanton of the H atom abstraction mechanisms computed for 160 K using the 12,11-CASSCF/cc-pVDZ-pp level of theory (black line). The red lines show the 12,11-NEVPT2/def2-TZVPP potential energy computed by single points along the respective paths.

Unimolecular instanton reaction rate constants

The rate constants reported in this work and presented in the main text are unimolecular, *i.e.,* represent the reaction of a pre-reactive complex [$H_2\cdots$IPy].

Table S7.

Unimolecular rate constants in s^{-1} of the two different mechanisms (concerted H_2 activation and H atom abstraction) computed with instanton theory. CASSCF: regular instanton theory computed using the CASSCF/cc-pVDZ-pp level of theory. Dual: Potential energy of instanton and reactant state corrected by NEVPT2/def2-TZVPP single point energy calculations.

	concerted H_2 Activation		H atom abstraction	
T/K	CASSCF	Dual	CASSCF	Dual
320			$2.06 \cdot 10^{+00}$	$2.80 \cdot 10+^{03}$
300			$6.12 \cdot 10^{-01}$	$5.17 \cdot 10^{+02}$
280			$2.07 \cdot 10^{-01}$	$1.28 \cdot 10^{+02}$
250			$3.89 \cdot 10^{-02}$	$1.77 \cdot 10^{+01}$
200			$2.15 \cdot 10^{-03}$	$6.08 \cdot 10^{-01}$
180	$9.17 \cdot 10^{-06}$	$1.38 \cdot 10^{+03}$	$6.46 \cdot 10^{-04}$	$1.55 \cdot 10^{-01}$
175	$4.87 \cdot 10^{-06}$	$3.59 \cdot 10^{+02}$		
170	$2.68 \cdot 10^{-06}$	$1.30 \cdot 10^{+02}$		
160	$8.79 \cdot 10^{-07}$	$2.39 \cdot 10^{+01}$	$1.92 \cdot 10^{-04}$	$3.82 \cdot 10^{-02}$
150	$3.43 \cdot 10^{-07}$	$5.23 \cdot 10^{+00}$		
145	$2.48 \cdot 10^{-07}$	$2.66 \cdot 10^{+00}$		
140			$5.60 \cdot 10^{-05}$	$9.07 \cdot 10^{-03}$
130			$3.02 \cdot 10^{-05}$	$4.30 \cdot 10^{-03}$
120			$1.63 \cdot 10^{-05}$	$2.08 \cdot 10^{-03}$
110			$8.92 \cdot 10^{-06}$	$1.00 \cdot 10^{-03}$
100			$4.90 \cdot 10^{-06}$	$4.70 \cdot 10^{-04}$
90			$2.62 \cdot 10^{-06}$	$2.06 \cdot 10^{-04}$
80			$1.45 \cdot 10^{-06}$	$1.00 \cdot 10^{-04}$
75			$1.06 \cdot 10^{-06}$	$6.62 \cdot 10^{-05}$
70			$8.12 \cdot 10^{-07}$	$4.83 \cdot 10^{-05}$
65			$5.74 \cdot 10^{-07}$	$3.26 \cdot 10^{-05}$
60			$4.57 \cdot 10^{-07}$	$2.23 \cdot 10^{-05}$
55			$3.11 \cdot 10^{-07}$	$1.60 \cdot 10^{-05}$
50			$2.49 \cdot 10^{-07}$	$1.07 \cdot 10^{-05}$

The resulting rate constants were fitted to the rate expression (1) given by Zheng *et al.* [2] (see Table S8):

$$k = A\left(\frac{T + T_0}{300\ \text{K}}\right)^n \times \exp\left(-\frac{E}{\text{R}}\frac{(T + T_0)}{(T^2 + T_0^2)}\right)$$

Please keep in mind that the parameter *A, n, E*, and T_0 represent mere fitting parameters rather than containing true physical information:

Table S8.
Paramters obtained by the fit in equation (1) using a least-squared-fitting routing implemented in Python 3.9.7.

	A in s^{-1}	n	E in J mol^{-1}	T_0 in K
H_2-Activation	2.022 x 10^{-8}	71.7	4806.9	257.8
H-Abstraction	2.190 x 10^{-8}	38.8	22674.0	381.6

6. References

(1) Das, P.; Bahou, M.; Lee, Y.-P., Reactions between atomic chlorine and pyridine in solid *para*-hydrogen: Infrared spectrum of the 1-chloropyridinyl (C_5H_5N−Cl) radical. *J. Chem. Phys.* **2013,** *138*.

(2) Zheng, J.; Seal, P.; Truhlar, D. G., Role of conformational structures and torsional anharmonicity in controlling chemical reaction rates and relative yields: butanal + HO_2 reactions. *Chem. Sci.* **2013,** *4*, 200-212.
